# Associations of Dietary Intake and Nutrient Status with Micronutrient and Lipid Composition in Breast Milk of Donor Women

**DOI:** 10.3390/nu15153486

**Published:** 2023-08-07

**Authors:** Noelia Ureta-Velasco, Adriana Montealegre-Pomar, Kristin Keller, Diana Escuder-Vieco, Javier Fontecha, María V. Calvo, Javier Megino-Tello, José C. E. Serrano, Nadia Raquel García-Lara, Carmen R. Pallás-Alonso

**Affiliations:** 1 Department of Neonatology, 12 de Octubre University Hospital, 28041 Madrid, Spain; nadiaraquelg.nrgl@gmail.com (N.R.G.-L.); kpallas.hdoc@gmail.com (C.R.P.-A.); 2 Research Institute i+12, 12 de Octubre University Hospital, 28041 Madrid, Spain; biol.kristin.keller@gmail.com (K.K.); diana.e.vieco@gmail.com (D.E.-V.); 3 Faculty of Medicine, Complutense University of Madrid, 28040 Madrid, Spain; 4 Epidemiología Clínica, Pontificia Universidad Javeriana, Hospital Universitario San Ignacio, Bogotá 110231, Colombia; montealegrepomar@gmail.com; 5 “Aladina-MGU”—Regional Human Milk Bank, 12 de Octubre University Hospital, 28041 Madrid, Spain; 6 Food Lipid Biomarkers and Health Group, Institute of Food Science Research (CIAL), CSIC-UAM, 28049 Madrid, Spain; j.fontecha@csic.es (J.F.); mv.calvo@csic.es (M.V.C.); jamegtel@gmail.com (J.M.-T.); 7 Department of Experimental Medicine, Faculty of Medicine, University of Lleida, 25008 Lleida, Spain; jceserrano@mex.udl.cat

**Keywords:** breast milk, human milk bank, associations, donors, diet, nutritional status, lipid profile, vitamins, minerals, docosahexaenoic acid

## Abstract

The influence of the diet and nutritional status of milk donors on the nutritional composition of donor human milk (DHM) is unknown. The present study aimed to determine the nutritional profile of DHM and the associations between donors’ dietary intake and nutritional status and the micronutrient and lipid composition in DHM. For this purpose, 113 donors completed a food frequency questionnaire, provided a five-day weighed dietary record, and collected milk for five consecutive days. Nutrient determinations in donors’ erythrocytes, plasma, urine, and milk were performed. Multiple linear regressions were conducted for the evaluation of the associations. We highlight the following results: DHM docosahexaenoic acid (DHA) was positively associated with donors’ plasma DHA content and donors’ DHA intake (R^2^ 0.45, *p* < 0.001). For every 1 g/day DHA intake, an increase of 0.38% in DHA content and 0.78% in total omega-3 content was observed in DHM (R^2^ 0.29, *p* < 0.001). DHM saturated fatty acids were positively associated with erythrocyte dimethyl acetals, plasma stearic acid, *trans* fatty acids intake, and breastfeeding duration and negatively associated with erythrocyte margaroleic acid (R^2^ 0.34, *p* < 0.01). DHM cholecalciferol was associated with plasma cholecalciferol levels and dairy intake (R^2^ 0.57, *p* < 0.01). Other weaker associations were found for free thiamin, free riboflavin, pyridoxal, dehydroascorbic acid, and the lipid profile in DHM. In conclusion, the diet and nutritional status of donors influence the fatty acid profile and micronutrient content of DHM.

## 1. Introduction

Donor human milk (DHM) is considered the best feeding option for preterm infants when their mother’s own milk is not available. Its use is increasing in neonatal units, primarily for infants at high risk of developing necrotizing enterocolitis or feeding intolerance, such as those weighing <1500 g and/or those at less than 32 weeks of gestational age (GA), infants with congenital heart disease, or those with severe intestinal disorders [1,2,3,4,5]. These infants are in a situation of “nutritional emergency”; therefore, knowledge of the essential nutrients in DHM and research into the factors contributing to its composition should be a priority.

According to studies carried out in different geographical areas of the world, there is evidence that the content of fatty acids (FAs) and some micronutrients in human milk (HM) depends on the maternal deposits of each nutrient at the end of gestation and maternal intake during the lactation period [6,7,8,9]. However, most studies have assessed the FA profile and information on micronutrients is much more limited.

In this context, micronutrients can be classified in a simplified way into two groups with respect to their secretion patterns in milk in relation to maternal intake and status and the response to supplementation. In general, water-soluble vitamins (except folate), fat-soluble vitamins, iodine, and selenium are considered to belong to a group of micronutrients for which secretion into HM is dependent on maternal intake or status, and maternal supplementation may increase their concentrations in milk [8,9,10,11]. Accordingly, iodine, vitamin A, thiamin, riboflavin, vitamin B6, and cobalamin have been established as priority nutrients for breastfeeding women [12]. The behavior of vitamin E is somewhat different, as neither plasma nor serum concentrations nor the usual maternal diet affect the vitamin E concentration in HM, although vitamin E supplementation has been shown to increase colostrum alpha-tocopherol levels [7,13]. On the other hand, the concentrations of folate and other minerals and trace elements in HM remain fairly constant regardless of maternal intake or status, although maternal stores may be affected if the intake of these nutrients is insufficient; maternal supplementation with the nutrients of this second group is particularly beneficial for the mother rather than for her infant [8,9,10,11,12]. 

Nevertheless, the results of the different surveys have not always been concordant [7], and the authors of two systematic reviews on this topic [8,9] highlighted the small sample sizes and the large heterogeneity in the studies. In addition, women’s diets vary over time and according to the country in which they live and their cultural environment [14,15,16,17]. For this reason, it is essential to have updated references in each country on the nutrients in HM.

Moreover, HM is a very complex and dynamic fluid, and its nutritional composition depends not only on maternal or infant factors but also on its chronobiology (i.e., it changes with the duration of lactation, the time of day, the length of time that elapses between feedings, the duration of feeding, and the circadian rhythm) [7,18,19,20,21]. All these factors can act as confounders when studying changes in HM content in relation to diet or maternal nutritional status and interfere with the interpretation of the data.

Furthermore, DHM requires its own studies as it is not comparable to breast milk in some respects [22]. Firstly, HM donors constitute a particular population for several reasons. Milk donors comprise a heterogeneous population of breastfeeding mothers in terms of the duration of pregnancy and stage of lactation. Studies carried out in Spain and other countries have shown how the sociodemographic characteristics of donor women differ from those of the general population [23,24]. Also, in addition to breastfeeding their own child, donors express surplus milk for donation, which might lead to increased maternal nutritional needs and a different nutritional milk profile. Secondly, DHM is subjected to procedures that may alter its nutritional profile, such as collection, mixing, multiple container changes, freeze–thaw cycles, storage, and pasteurization [22,25]. For example, a decrease in vitamins C and B6 in breast milk due to exposure to light and freezing has been observed, as well as a decrease in vitamins C and B6 and folate due to pasteurization [26,27,28,29,30]. A recent study found that, during long-term storage of human milk, refrigerated or frozen, the membrane phospholipids of milk fat globules were degraded and triacylglycerols were released from the core, with sphingomyelin being the glycerophospholipid showing the highest rate of lipolysis [31]. Typically, from extraction until it is administered to the recipient infants, DHM is exposed to light and undergoes two freeze–thaw cycles, as well as processing, usually involving Holder pasteurization [26,30]. Overall, data indicate that Holder pasteurization affects several components of milk but mainly bioactive and immunomodulatory, rather than nutritional, components [26]. Although clinical practices show that beneficial properties of DHM persist after Holder pasteurization, new pasteurization techniques are being investigated to improve the biological and nutritional quality of DHM [32,33]. Lastly, in 2020, Perrin published a systematic review on the nutritional composition of DHM, highlighting the need for future studies on the micronutrient content especially, as data on the vitamin and mineral composition of DHM were scarce [22].

Therefore, because of the great importance of nutrition in the development of very preterm or critically ill newborns, it is essential to have information on the nutritional composition of DHM and the factors that might impact it, such as diet, in order to improve our recommendations to donors. To the best of our knowledge, the only study in which a dietary assessment of milk donors was performed was a randomized, controlled trial about the effect of donors’ supplementation with docosahexaenoic acid (DHA) on the DHA content in DHM conducted in the United States [34].

Hence, the aim of this study was to determine, in a large population of donors from a milk bank in Madrid (Spain), the macronutrient composition, lipid profile (fatty acid profile, lipid classes, relative composition of phospholipids, and molecular species of triacyclglycerols), and micronutrient composition (free thiamine, free riboflavin, nicotinamide, pyridoxal, pantothenic acid, folic acid, cobalamin, ascorbic acid, dehydroascorbic acid, retinol, α-tocopherol, γ-tocopherol, cholecalciferol, 25(OH)D_3_, iodine, calcium, phosphorous, and selenium) of raw DHM and their associations with donors’ dietary intake and nutrient status.

## 2. Materials and Methods

### 2.1. Study Design and Participants

This study was part of a larger research project, a cross-sectional observational study conducted at the Aladina MGU Regional Human Milk Bank (RHMB) at the “12 de Octubre” University Hospital in Madrid, Spain. Two previous articles based on this research project have recently been published [35,36].

The main aim of the present work was to study the correlations between the diet, nutritional status, and nutritional milk composition (macronutrients, lipids, water-soluble and fat-soluble vitamins, calcium, phosphorous, and selenium) of milk donors. For this purpose, we recruited donors by opportunity at the RHMB between August 2017 and February 2020. The inclusion criterion was to be an active donor in our RHMB (i.e., to have donated at least once in the last two months) without communication barriers. In our RHMB, the requirements to be a milk donor are that potential donors must have a healthy lifestyle, not be suffering from illnesses or taking any medications that are incompatible with the donation, be successfully breastfeeding their own child, and want to donate their surplus milk voluntarily and altruistically. Normally, milk donors are accepted from 3 weeks postpartum to ensure that breastfeeding is well established. There are no exclusion criteria regarding the age of the donor or the gestational age of her child, nor is there an upper limit on the length of breastfeeding. Women who wish to donate milk after the death of their baby, vegetarians or vegans with normal plasma cobalamin values committed to taking the recommended vitamin B12 supplements, and women with well-controlled hypothyroidism are accepted as donors. The statistical calculation for the sample size was undertaken in G*Power 3.1 [37]; for an effect size of 0.15 (moderate), an alpha of 0.05, power of 0.80, and 8–10 predictors at most, assuming losses of 5%, the sample size was 115 milk donors.

The study was carried out in agreement with Declaration of Helsinki and was approved by the Clinical Research Ethics Committee of the Hospital Universitario “12 de Octubre” (protocol code 15/269). All participants gave written informed consent. 

### 2.2. Study Protocol

The full study protocol and laboratory studies have been previously described in detail in the abovementioned studies [35,36].

**Figure 1:**
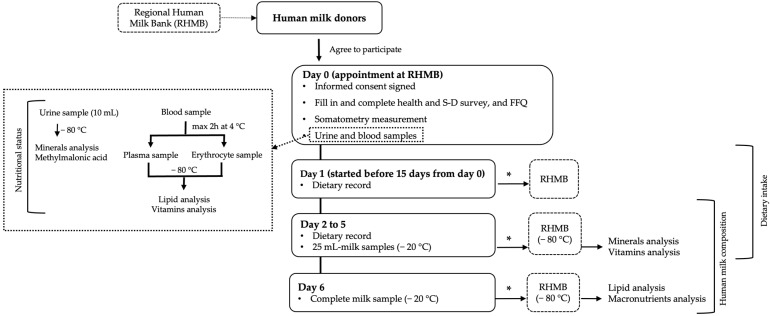
Study protocol flowchart. * Provided to the RHMB during the first 15 days after the end of the study. Abbreviations: S-D, sociodemographic; FFQ, food frequency questionnaire.

The study protocol is shown in Figure 1.

In summary, the study of participating donors comprised a health and sociodemographic survey; somatometric measurements; blood and urine determinations to study their nutritional status; a dietary study including a food frequency questionnaire (FFQ) and a five-day weighed dietary record, taking into account the intake of pharmacological nutrient supplements; and a detailed study of the nutritional composition of their milk. 

At the first visit to the RHMB (day 0), fasting urine and blood samples were collected. Plasma and red blood cells were obtained from the blood. All biological samples were frozen at −80 °C until analysis of the biochemical indicators of nutrition. In addition, milk donors provided their informed consent, underwent a somatometric study (weight, height, and body mass index calculation), filled in the health and sociodemographic survey, and completed a food consumption frequency questionnaire. Donors were trained in how to complete the five-day weighed dietary record and collect and keep frozen milk samples at home. They also were supplied with the required material.

Within the following 15 days after the visit to the RHMB, the five-day dietary recording and the collection of the five-day milk samples were carried out almost concomitantly for 6 days in a row, with milk collection starting 1 day after the start of the dietary recording and ending 1 day after the recording of the diet had been completed. We ensured that the DHM we analyzed met the same conditions and underwent the same processes as the raw milk donated to our RHMB. Therefore, in order to replicate the donors’ milk expression routine, participants were not asked to express milk at any particular time, the only requirement being complete emptying of one or both breasts, and the milk was collected in the same sterile transparent bottles that the donors use under normal donation conditions. From days 2 to 5, donors collected a 25 mL milk sample from each expression (at least one per day) and froze it at −20 °C. On day 6, they collected and froze one complete milk extraction. Donors were asked to deliver their dietary records and their frozen milk samples to the RHMB during the first 15 days following the end of the study. Donors transported the milk samples in isothermal containers with cold packs, and the milk remained frozen at the RHMB at −20 °C until thawing for aliquoting.

In the RHMB, different milk samples from the same day were mixed and aliquoted, but the milk from different days was not mixed, as the composition of the milk from each day was analyzed individually. 

Therefore, the milk samples that were analyzed went through a process of extraction, freezing at the donors’ homes, transport to the RHMB, and thawing to collect the aliquots, similar to the process that DHM usually undergoes at our RHMB. The milk samples were subsequently frozen until analysis in the respective laboratories at −80 ºC instead of −20 °C, as the freezing time was much longer than that of the DHM at the RHMB. Before analysis in the laboratory, the milk was thawed again. Thus, the studied milk underwent a total of two freeze–thaw cycles, just like the DHM in our RHMB, the only difference being that it was not pasteurized. 

The milk samples collected from days 2 to 5 were used for the study of vitamins and minerals, and the complete milk collection sample from day 6 was used for the integral characterization of the lipid fraction. The data on the intake of food, beverages, and pharmacological nutrient supplements obtained from the five-day dietary record were analyzed using DIAL^®^ software (DIAL.EXE Version 3, February 2014, Alce Ingeniería, Madrid, Spain) to calculate daily nutrient intakes [38]. Nutrient intakes were compared with the Recommended Dietary Allowances/Adequate Intakes provided by the American Institute of Medicine [39,40,41,42,43,44] and the Population Reference Intakes/Adequate Intakes provided by the European Food Safety Authority [45] for lactating women.

### 2.3. Nutrient Analysis

Lipid analyses were conducted by the Food Lipid Biomarkers and Health Group at the Institute of Food Science Research (CIAL, CSIC-UAM). Fatty acid methyl esters were analyzed by GC-MS in erythrocytes, plasma, and milk. After fat extraction from human milk samples from 20 randomly selected donors, the separation and quantification of lipid classes were performed using an HPLC evaporative light scattering detector (ELSD), and the determination of TAG molecular species was undertaken using GC-FID.

Vitamin and mineral analyses were conducted by the NUTREN-Nutrigenomics Group of the Department of Experimental Medicine at the University of Lleida, Spain. Micronutrient determinations were carried out using chromatographic analyses, mass spectrometry, and immunoassays. Free thiamin, free riboflavin, nicotinamide, pyridoxal, pantothenic acid, folic acid, cobalamin, ascorbic acid, dehydroascorbic acid, retinol, α-tocopherol, γ-tocopherol, cholecalciferol, 25(OH)D_3_, iodine, calcium, phosphorous, and selenium were determined in milk. The erythrocyte glutathione reductase activity coefficient (EGRAC), riboflavin, nicotinamide, pantothenic acid, pyridoxamine, and hemoglobin were determined in erythrocytes. The EGRAC, which assesses erythrocyte glutathione reductase activity in terms of an excess of flavine adenine dinucleotide compared to baseline activity, is a functional property used to determine the nutritional riboflavin status. Thiamin, riboflavin, nicotinamide, pantothenic acid, pyridoxine, pyridoxamine, folic acid, cobalamin, holotranscobalamin, homocysteine, ascorbic acid, retinol, 25(OH)D_3_, 1,25(OH)_2_D_3_, α-tocopherol, γ-tocopherol, triacylglycerols, total cholesterol, HDL-cholesterol, and LDL-cholesterol were determined in plasma. Creatinine, methylmalonic acid, iodine, sodium, calcium, and phosphorous were determined in urine. 

Determinations of macronutrients (total fat, proteins, and carbohydrates) in DHM were carried out at the RHMB using Fourier-transform mid-infrared (FT-MID) spectroscopy in a milk analyzer (MilkoScan FT2, FOSS S.A., Barcelona, Spain).

A full description of the analytic techniques employed for each of the nutrients is available in a previous paper [36], as mentioned above.

### 2.4. Statistics

Statistical analysis was performed with the STATA 14 program.

For descriptive analysis, qualitative variables were presented as absolute and relative frequencies and quantitative variables as medians and the interquartile range or as means and the standard deviation depending on their distribution, which was established by performing the Shapiro–Wilk normality test. 

For the multivariate analysis of associations, multiple linear regressions (MLRs) were performed using each of the lipids, macro- and micronutrients in DHM as dependent variables, except for iodine, which had been previously studied [35]. For vitamins and minerals, the average of the four-day milk content of each participant was used. The independent variables were as follows: the clinical and sociodemographic characteristics; the somatometric study results; the levels of nutrients in donors’ plasma, erythrocytes, and urine; the results for intake from the FFQ; and the average intake of nutrients recorded by donors in the five-day dietary record. The independent variables with significant associations (*p* < 0.05) with milk content in the bivariate analysis were entered into the models. Once the independent variables and their probable interactions were introduced in each of the models, the variables with *p* > 0.05 were removed, and confounding variables were evaluated until the most parsimonious model was obtained. Each model was diagnosed, and some outliers were removed to improve the fit. For each model, regression diagnosis was performed with the residuals; the assumptions of linearity with each independent variable were assessed with scatter plots and, in addition, homoscedasticity of variance was assessed with the Breusch–Pagan test and normal distribution with p–p plots. The independence of observations was also assessed with vif and omitted variable bias was tested with ovtest. The following diagnostic plots were used for the influential points: residual versus fitted values (rvfplot), augmented component-plus-residual plots (acpr plots), leverage against normalized squared residuals (lvr2plot), and Cooks’ distance. In the models’ tables, the influencing points identified and removed for each model after individual analyses were recorded.

## 3. Results

### 3.1. Population Studied

A total of 114 milk donors were recruited: 112 were omnivores (93 with full-term infants, 16 with preterm infants who were not hospitalized, and 3 with preterm infants <32 weeks of gestational age and/or with <1500 g birthweight hospitalized in the neonatology service) and 2 were ovo-lactovegetarians (1 with a preterm infant 36^+4^ weeks of gestational age and the other with a full-term infant). One of the omnivore donors left the study early; thus, complete data were obtained from 113 donor women.

Table 1, Table 2, Table 3 and Table 4 show the results of the sociodemographic and health survey, including the characteristics of the donors’ breastfed infants and breastfeeding habits.

The median age of donors was 35.6 years and they had a median body mass index (BMI) of 22.9 kg/m^2^, similar to their pre-pregnancy BMI (22.1 kg/m^2^). BMI at the time of the study was classified as normal for 71% of the sample (Table 1). In terms of dietary habits, 29% reported having changed eating habits in the last two years towards a healthier diet or due to changes in dairy consumption. There were 18 donors with some type of food restriction: dairy (*n* = 6), fish (*n* = 3), meat (*n* = 3), eggs (*n* = 2), nuts (*n* = 1), and others (*n* = 3). In total, 87 of the 109 donors who specified the type of salt consumed (79.8%) reported taking iodized salt.

Fifty-five percent of donors reported having no previous children. Four donors had twin pregnancies, among whom two lost a fetus (one ovo-lactovegetarian donor with a term infant and one omnivore donor with a preterm infant 24^+4^ weeks of gestational age) and one lost one of her twins at 27^+0^ weeks of gestational age during the first week of life. Another donor had one fetal death at 22^+6^ weeks of gestational age 2 months previously (Table 2). 

Five percent of donors were breastfeeding more than one child (tandem or twin breastfeeding) at the time of the study. The median gestational age at birth was 39^+4^ weeks, and 47% of children were male. Regarding the 19 donors with preterm deliveries, the post-menstrual/corrected age of the infants ranged from 30^+6^ weeks to 17.5 months. Seventy-two percent of mothers employed simple electrical breast pumps (Table 3 and Table 4).

Regarding nutrient supplementation, 112 of 113 donors (99.1%) reported consuming pharmacological nutritional supplements during pregnancy and 102 of 113 donors (90.3%) during lactation. Most participants took supplements of folic acid, vitamin B12, and iodine during pregnancy. The number of donors who took supplements of each nutrient, both during gestation and lactation, and the median daily dose that they consumed are available in Table S1 in the Supplementary Material.

### 3.2. Diet Survey and Nutritional Status

The results of the five-day dietary record, the FFQ, and the analysis of nutrients in the erythrocytes, plasma, and urine of the donors for the assessment of their nutritional status are displayed as supplementary data (Tables S2–S7). References [36,48,49,50,51,52,53,54,55,56,57,58,59,60,61,62,63,64,65,66,67,68,69,70,71,72] are cited in the Supplementary Materials.

### 3.3. Composition of Donor Human Milk

Data on the nutritional composition of the DHM are presented in Table 5, Table 6 and Table 7. Table 5 presents the macronutrient composition, the lipid classes’ profile, the relative composition of phospholipids, and the molecular species of triacylglycerols in the DHM. Table 6 presents the concentrations of 30 FAs, and Table 7 shows the concentrations of 15 micronutrients in the DHM.

### 3.4. Associations between Clinical and Somatometric Characteristics, Donors’ Daily Nutrient Intake, Their Plasma and Erythrocyte Nutrient Levels, and Nutrient Levels in DHM: Multivariate Analysis

Models in which significant associations were found are described below.

#### 3.4.1. Lipid Associations in DHM

Levels of FAs in DHM are expressed as percentages of fat (Table 8).

The percentage of total saturated fatty acids (SFAs) in DHM was associated with the donors’ erythrocyte deposits of dimethyl acetals (DMAs) and C17:1 (margaroleic acid), plasma C18:0 (stearic acid) levels, *trans* fatty acids (TFAs) consumed in the dietary record, and breastfeeding time. For each 1% increase in DMA in erythrocytes, adjusted SFA levels in DHM increased by 2.7%. In addition, for each 1% increase in plasma C18:0 levels, the adjusted SFA content in DHM increased by 2%, and for each 0.1% of kcals. consumed from TFA in the dietary record, the adjusted percentage of SFAs in DHM increased by almost 0.6%. On the other hand, for each 0.1% increase in levels of erythrocyte C17:1, adjusted SFA levels in DHM decreased by approximately 1.4%. Finally, for each month of breastfeeding time, the adjusted percentage of SFA in DHM increased by 0.2%.

The amount of monounsaturated fatty acids (MUFAs) in DHM was positively associated with donors’ plasma MUFA levels and erythrocyte C17:1 levels. Adjusted MUFA levels in DHM increased by approximately 0.5% per 1% increase in plasma MUFAs and by almost 1.4% for each 0.1% increase in the erythrocyte C17:1 level.

PUFA levels in DHM were weakly associated with donors’ daily average PUFA intake according to the five-day dietary record. For each 5 g of PUFAs in the daily diet, PUFAs in DHM increased by nearly 0.7%.

A positive association was found between linoleic acid levels in DHM and the linoleic acid levels in donors’ erythrocytes and average intake of meat, fish, and eggs in the five-day dietary record. For every 1% increase in the percentage of linoleic acid in erythrocytes, adjusted levels in DHM increased by 0.6%. Moreover, for each daily serving of meat, fish, or eggs consumed, the adjusted percentage of linoleic acid in DHM increased by 0.7%. For linolenic acid (C18:3-n3), no significant associations were found between donor clinical and somatometric characteristics, donor plasma/erythrocyte levels or breastfeeding characteristics and DHM content.

DHA levels in DHM were associated with DHA donors’ plasma levels and the average daily DHA intake according to the five-day dietary record. For every 1% increase in DHA in donors’ plasma, the percentage of DHA in DHM increased by approximately 0.2%, and for every 1 g of daily DHA intake, the adjusted percentage of DHA in DHM increased by almost 0.4%. 

The association between total omega-3 in DHM and donors’ DHA intake was positive and significant. For every 1 g of DHA per day in the five-day dietary record, the percentage of omega-3 in DHM increased by 0.8%.

#### 3.4.2. Vitamin, Mineral, and Trace Element Associations in DHM

##### Water-Soluble Vitamins (Table 9)

Associations were found between free thiamin levels in DHM and donors’ thiamin plasma levels, daily dairy intake in the five-day dietary record, and breastfeeding time.

For each 0.1 mcg/L of plasma thiamin, adjusted free thiamin levels in DHM increased by 1.1 mcg/L. On the other hand, for each dairy serving/day from the dietary record, adjusted free thiamin levels in DHM decreased by 2.9 mcg/L, and for each month of lactation, thiamin levels in DHM decreased by 0.5 mcg/L.

Regarding riboflavin, a positive association was found between DHM-free riboflavin and the daily intake of riboflavin in the five-day dietary record, as well as receiving vitamin B2 supplementation during lactation. For 1 mg/day of riboflavin intake, the adjusted levels of free riboflavin in DHM increased by 21 mcg/L, and if donors received riboflavin supplementation during lactation, the adjusted levels in DHM increased by approximately 40 mcg/L.

In relation to pyridoxal levels in DHM, associations were observed with respect to vitamin B6 supplementation during pregnancy and breastfeeding time. If donors received vitamin B6 supplements during pregnancy, adjusted pyridoxal levels in DHM increased by 11.6 mcg/L, and for each month of breastfeeding, adjusted pyridoxal levels in DHM decreased by 0.8 mcg/L.

No significant associations were found for nicotinamide, pantothenic acid, folic acid, or cobalamin levels in DHM.

There were associations between dehydroascorbic acid in DHM and donors’ plasma ascorbic acid levels, average daily fruit intake in the dietary record, and breastfeeding time. For each average daily serving of fruit from the five-day dietary record, adjusted dehydroascorbic acid levels in DHM increased by 0.3 mg/dL. Breastfeeding time and plasma ascorbic acid levels were negatively associated with dehydroascorbic acid levels in DHM, but the adjusted values were very low.

##### Fat-Soluble Vitamins (Table 9)

There was a positive association between cholecalciferol levels in DHM, donors’ cholecalciferol plasma levels, and daily dairy intake. For each 10 pg/mL of plasma cholecalciferol, adjusted cholecalciferol levels in DHM increased by 173 pg/mL. According to the five-day dietary record, for each average daily serving of milk and dairy products, adjusted cholecalciferol levels in DHM increased by 521 pg/mL, equivalent to almost 21 UI/L.

There was a positive association between 25(OH)D_3_ in DHM and donors’ 1,25(OH)_2_D_3_ plasma levels, vitamin D supplementation, and breastfeeding time. For each 10 pg/mL of 1,25(OH)_2_D_3_ in donors’ plasma, 25(OH)D_3_ in DHM increased by 1.6 pg/mL; in addition, if donors received vitamin D supplementation during lactation, 25(OH)D_3_ in DHM increased by 35.7 pg/mL, and for each month of breastfeeding, 25(OH)D_3_ in DHM increased by 2.2 pg/mL.

##### Minerals and Trace Elements

The association found between iodine levels in DHM and iodine supplementation was described in a previous publication [25]. No significant associations were found for selenium, calcium, or phosphorus levels in DHM.

**Table 9:** Multivariate analysis of vitamins, minerals, and trace elements in donor human milk.

Vitamins	Associated Variables	Beta	SE	t	P > |t|	95% CI
Free thiamin, B1 (mcg/L) Observations = 113 R^2^ = 0.12	Plasma thiamin (mcg/L)	10.817	4.915	2.20	0.030	[1.075, 20.559]
	Milk and dairy products (average servings/day from the dietary record)	−2.880	1.090	−2.64	0.009	[−5.040, −0.720]
	Breastfeeding time (months)	−0.494	0.210	−2.35	0.021	[−0.911, −0.077]
Free riboflavin, B2 (mcg/L) ^a^ Observations = 100 R^2^ = 0.21	Riboflavin intake (average mg/day from the dietary record)	20.991	8.758	2.40	0.018	[3.609, 38.373]
	Vitamin B2 supplementation during lactation (receiving or not)	39.742	15.258	2.60	0.011	[9.459, 70.024]
Pyridoxal, B6 (mcg/L) ^b^ Observations = 106 R^2^ = 0.17	Vitamin B6 supplementation during pregnancy (receiving or not)	11.629	3.114	3.73	<0.001	[5.454, 17.804]
	Breastfeeding time (months)	−0.776	0.273	−2.84	0.005	[−1.318, −0.234]
Dehydroascorbic acid (mg/dL) Observations = 112 R^2^ = 0.15	Plasma ascorbic acid (mcM)	−0.014	0.005	−2.92	0.004	[−0.024, −0.005]
	Fruits (average servings/day from the dietary record)	0.271	0.128	2.12	0.036	[0.018, 0.524]
	Breastfeeding time (months)	−0.062	0.025	−2.47	0.015	[−0.112, −0.012]
Cholecalciferol (pg/mL) ^c^ Observations = 32 R^2^ = 0.57	Plasma cholecalciferol (pg/mL)	17.342	4.238	4.09	<0.001	[3.178, 23.510]
	Milk and dairy products (average servings/day from the dietary record)	520.533	168.819	3.08	0.005	[174.722, 866.343]
25(OH)D_3_ (pg/mL) ^d^ Observations = 99 R^2^ = 0.21	Plasma 1,25(OH)_2_ D_3_ (pg/mL)	0.156	0.042	3.73	<0.001	[0.073, 0.238]
	Vitamin D supplementation during lactation (receiving or not)	35.673	12.569	2.84	0.006	[10.720, 60.625]
	Breastfeeding time (months)	2.225	1.076	2.07	0.041	[0.089, 4.360]

^a^ After a diagnosis of the model, the data entries 110 and 51 were removed. ^b^ After a diagnosis of the model, the data entries 2, 32, 37, 46, 55, and 110 were removed. ^c^ Adjusted for the average amount of vitamin D supplementation from the dietary record. After a diagnosis of the model, the data entries 22, 26, 40, 41, 53, 102, and 115 were removed. ^d^ Adjusted for vitamin D supplementation during gestation (receiving or not). After diagnosis of the model, the data entries 4, 32, and 102 were removed. Abbreviations: SE, standard error.

#### 3.4.3. Macronutrients Associations in DHM

No associations were found between carbohydrate and protein content in DHM and donors’ clinical and somatometric characteristics, nutritional biochemical determinations, or dietary intake.

## 4. Discussion

This cross-sectional study investigated the associations between macronutrients, the fatty acid profile, lipid classes, the relative composition of phospholipids, molecular species of triacyclglycerols, and 14 micronutrients (free thiamine; free riboflavin; nicotinamide; pyridoxal; pantothenic acid; folic acid; cobalamin; vitamins C, A, D, and E; calcium; phosphorous; and selenium) in raw DHM and the diet, as well as the nutritional status of 113 milk donors from the Regional Human Milk Bank in Madrid, Spain. To the best of our knowledge, this is the first study in which these associations have been evaluated in donor human milk. For this purpose, donors completed an FFQ, provided a five-day weighed dietary record, and collected milk for 5 consecutive days. In addition, somatometric measurements and nutrient determinations in donors’ erythrocytes, plasma, urine, and milk were performed. 

One of the most important findings of the present study was the correlation between donors’ DHA intake and DHA plasma levels and the DHA content of the raw DHM. This result is important because, in a recent study [91], the DHA levels in DHM were lower than in the mother’s own milk for very preterm infants, indicating that DHM provides an insufficient supply of DHA to these patients, who comprise an already DHA-deficient population [92]. Our results are consistent with those of the only study that has determined the DHA content of DHM in relation to the DHA intake of milk donors. In that randomized, controlled trial conducted in Ohio and published in 2013 [34], milk donors supplemented with an algal-derived product providing a DHA dose of 1 g/day had significantly higher DHA content in their milk than baseline samples, and DHA content was over four times higher than the placebo group after 14 days of supplementation. However, the sample size was very small, and the DHA intake at baseline was only 23 mg/day. In our observational study, the median donor DHA intake was 310 mg/day, and for every 1 g/day of DHA intake, the adjusted DHA content in DHM increased by 0.38 g/100 g fat. This increase was somewhat smaller than that reported by Makrides in 1996 [93] in a randomized study of maternal supplementation (lactating non-donor mothers) with preformed algae-derived DHA at different doses, where the DHA content of breast milk increased by approximately 0.1 g/100 g fat for each 0.1 g of DHA intake. In this sense, our approach adds information with respect to the real operating conditions of a milk bank without modifying the dietary and supplementation habits of the donors. As pasteurization has not been shown to significantly impair the DHA content of DHM [26,94,95], it could be inferred that increasing donors’ DHA intake is a feasible strategy to achieve a higher DHA content in pasteurized DHM. In addition, we found a positive association between DHA intake and the percentage of omega-3 in the DHM. On the other hand, we found no association between the content of DHA in DHM and the intake of its precursors, such as ⍺-linolenic acid. In a previous study, supplementing mothers with flaxseed oil, which is very rich in linolenic acid, did not enhance the DHA content of their milk [96]. Enhancement of donors’ DHA intake can be achieved by both increasing oily fish intake and/or pharmacological supplementation [97]. In our previous study comparing the nutritional milk composition of vegetarian or vegan lactating women vs. omnivore human milk donors, the milk DHA content was lower by half in the vegetarian/vegan women group as a result of their lower intake of DHA [36]. The availability of DHA supplements from algal oil offers vegetarian or vegan donors the possibility of increasing their DHA intake from a plant-based source. Supplementation with 250 mg of algae-derived DHA daily has been shown to increase plasma DHA content in omnivores, vegetarians, and vegans [98], and the use of a DHA/EPA supplement was positively associated with the DHA content in milk from lactating women following vegan, vegetarian, and omnivore diets [99]. Thus, with the evidence available at present, it could be deduced that one method for increasing the DHA content of DHM from vegetarian/vegan mothers is the intake of algae-derived DHA supplements; however, to the best of our knowledge, no randomized controlled study has been carried out to confirm this.

We also found a positive correlation between maternal PUFA intake and the PUFA content in DHM. This positive correlation was found in a previous work that studied the PUFA content in breast milk of non-donor lactating mothers during the first month of lactation [100]. 

It is remarkable that other correlations found in this study regarding lipid content have not been identified before. The DHM linoleic acid content increased with the erythrocyte linoleic acid content, as well as, interestingly, with combined intake of meat, fish, and eggs. The proportion of DHM MUFAs increased with plasma MUFA levels and erythrocyte margaroleic acid levels. The proportion of DHM SFAs increased with increased erythrocyte DMA content, plasma stearic acid, TFA intake, and breastfeeding time, but decreased with increased erythrocyte margaroleic acid content. 

In terms of micronutrients, various associations were found between the donors’ diet/nutritional status and the DHM composition. 

Regarding B-group vitamins, we found associations with the content of free thiamin, free riboflavin, and pyridoxal in milk. We were not able to demonstrate positive associations between the milk content of other B-group vitamins and maternal intake or erythrocyte/plasma levels, although all of them (except folate) are considered to be dependent on diet and maternal stores [11]. 

Free thiamin in DHM was positively correlated with plasma thiamin levels and negatively associated with breastfeeding time and the daily intake of dairy. Free riboflavin in DHM was positively associated with riboflavin intake and vitamin B2 supplementation during lactation. Pyridoxal in DHM was positively associated with vitamin B6 supplementation during pregnancy and negatively associated with breastfeeding time. In the literature, the concentrations of these three vitamins in human milk are strongly associated with maternal intake [7,8,9,101]. However, the influence of maternal stores on milk content depends on whether the mother has adequate or poor status in the case of thiamin, while it is controversial in the case of riboflavin and positive in the case of vitamin B6 [7]. Our donor population showed low plasma thiamin levels and 28% showed riboflavin deficiency according to the erythrocyte glutathione reductase activity coefficient study, but, nevertheless, the population showed adequate levels of plasma riboflavin. We were unable to assess the vitamin B6 nutritional status of donors because we did not determine plasma pyridoxal phosphate. In terms of diet, the median intakes of vitamins B1, B2, and B6 were above the recommended values, and the prevalence of inadequate intake was 6.2%, 15.9%, and 1.8%, respectively. The content of these three vitamins in the DHM can be considered low compared to previously published data [75,76,79] and considering that free thiamin should comprise about 30% of the total thiamin content in HM [7], free riboflavin should comprise about 39% of the total riboflavin content, and pyridoxal is the predominant form of vitamin B6 in breast milk [10]. In addition, both plasma nicotinamide levels and milk levels in our donor population were low [77], despite adequate niacin intake in all the donors studied. It remains uncertain whether the low content of free thiamin, free riboflavin, nicotinamide, and pyridoxal in DHM was due to deficient maternal status; prolonged average breastfeeding time; or the depletion of vitamins exposed to photodegradation, freezing, and storage. On the other hand, the levels of cobalamin in DHM were adequate with respect to the reference values for HM [80], and donors’ intake and plasma levels for this micronutrient were also adequate. Available evidence indicates a positive correlation between maternal cobalamin intake and milk vitamin B12 concentration in women with deficient B12 intake or depleted B12 stores [7,9,102]. It is possible that, in a population such as ours with adequate cobalamin intake and stores, the usual diet does not affect the concentration of cobalamin in milk. 

Donors’ fruit consumption was associated with dehydroascorbic acid content in DHM. One of the most consistent findings of the two systematic reviews conducted on the effect of maternal diet on the nutritional composition of breast milk [8,9] was the influence of maternal vitamin C intake on its content in milk, although the total number of studies in which this result was found was only three [101,103,104]. Interestingly, the study published by Hoppu in 2005 [104] reported that the vitamin C concentration in the milk of mothers with atopic disease was associated with their vitamin C intake but not with vitamin C supplementation. We were also unable to detect an association between vitamin C supplementation and vitamin C content in DHM. It should be noted that both our study and Hoppu’s were observational studies in which the rates of the use of vitamin C-containing supplements were 43.5% and 50% and the median daily doses of vitamin C supplementation were 80 mg and 75 mg, respectively.

For fat-soluble vitamins, associations were found only with vitamin D. The vitamin D content of the DHM was similar to the usual values for vitamin D in breast milk, widely known to be low for the needs of the infant [83]. It was noteworthy that the donor population studied showed a high percentage of vitamin D deficiency (87.7%). The content of cholecalciferol in DHM was positively correlated with the plasma cholecalciferol levels (but not with plasma 25(OH)D_3_ levels) and the daily intake of dairy. Furthermore, the content of 25(OH)D_3_ in DHM increased with the use of vitamin D supplementation during breastfeeding. These findings are in line with observations from previous studies [7,9]. Neither retinol nor vitamin E in DHM were associated with donors’ intake or status. In the case of vitamin A, donors’ stores were adequate, and it has been reported that both maternal intake and maternal status influence milk content when maternal stores are depleted [7]. The α-tocopherol content of DHM was adequate in relation to the reference values for HM [84], although the status of the donors was deficient. In general, neither maternal stores nor dietary intake of vitamin E influence HM vitamin E concentrations, although vitamin E supplementation appears to increase colostrum alpha-tocopherol levels [7,13]. 

Finally, we found no association between the carbohydrates, proteins, lipid classes, phospholipids, molecular species of triacylglycerols, calcium, phosphorus, and selenium content in the DHM and the independent variables studied. The selenium content in DHM was not associated with dietary intake in the present study, although it is known that the diet influences selenium HM content [105]. The calcium and selenium content in DHM was low and phosphorus content was adequate according to the reference values for HM [79,87,88,89]. 

In summary, we studied the factors that might influence the nutritional content of DHM, and we highlight the following as strengths: the detailed study of the supplementation habits of donors since gestation, the five-day weighed dietary record, and the determination of nutrients in donors’ erythrocytes, plasma, and urine. In addition, the dietary study was carried out concomitantly with the collection of milk over five consecutive days. Milk from different donors or different days was not pooled to allow us to determine associations between the content of each nutrient in DHM and donors’ intake, status, and characteristics. However, our study has some limitations. It is important to point out that the studied DHM comprised raw milk, and although the impact of pasteurization on the nutrient content in DHM has been previously studied [26,33], we do not know if all the associations observed would remain after the pasteurization of milk. Holder pasteurization is the most widely used heat treatment and the most studied. According to a review on the effect of Holder pasteurization [26], vitamin C (ascorbic acid + dehydroascorbic acid) and vitamin B6 decrease significantly with Holder pasteurization, while vitamins D, B2, B3, B5, and B12, as well as biotin, do not seem to be affected. Vitamins A and E have shown different responses to Holder pasteurization in different studies. The total lipid content is preserved after Holder pasteurization, as well as the fatty acid composition. In particular, DHA is not affected by Holder pasteurization [26,94,95]. New types of processing for DHM are being investigated in order to preserve as many of its properties as possible, with different results in terms of the degree of retention and loss of different nutrients [32,33]. As a next step, it would be interesting to study the relationship between the diet and nutritional status of donors and the nutrient content of the processed milk as the final product to be given to the recipient infants. Another limitation of our study was that, although we did standardize milk collection in terms of the type of expression (full expression), we did not standardize milk collection in terms of the time of milk expression or the time that had elapsed since the previous feeding or expression, as recommended [106]. We also did not protect photosensitive vitamins in milk from light. The great heterogeneity in the population studied in terms of breastfeeding time and duration of gestation was also noteworthy. It is known that all these factors influence the nutrients in milk. However, we wanted to reproduce the reality of our human milk bank. On the other hand, our results may not be generalizable to contexts other than a human milk bank. Although we controlled for an important number of covariates that could have acted as confounders, the large number of factors that can modify the nutritional content of human milk, as well as the complexity of nutrient metabolism—which is also influenced by the genetics of the mother–infant dyad itself—makes it very difficult to study interactions between maternal diet and milk composition. The interpretation of the results of biochemical nutritional indicators in breastfeeding mothers is also difficult due to the limited information available for this population and even more so in the case of prolonged breastfeeding [107]. A longitudinal study design could have provided additional information on changes in nutrient stores throughout lactation and their possible association with the composition of DHM. Also, given that the behavior of some nutrients in human milk with respect to the maternal diet may differ depending on the socioeconomic status of lactating mothers, access to food, and maternal nutritional status, the findings from our population of donors, who exhibited a high educational level and lived in a developed country, may not be generalizable to other environments. 

However, we consider that the results obtained in this study are relevant since we have shown that there are associations between the nutritional composition in raw DHM and the diet, as well as the nutrient deposits of donors—despite the heterogeneity among the donors regarding the duration of pregnancy and breastfeeding time—and the procedures to which raw DHM is subjected, such as extraction, freezing, mixing, and storage in transparent glass containers. 

## 5. Conclusions

In conclusion, the diet and nutritional status of donors influence the fatty acid profile and some of the micronutrients in the raw DHM. One of the most important results was the positive correlation found between the DHA content in the DHM and both the DHA intake and the plasma DHA levels of the donors. In our study, for every 1 g/day of DHA intake, an increase of 0.38% in DHA content and 0.78% in total omega-3 content was observed in DHM. DHM saturated fatty acids were positively associated with erythrocyte dimethyl acetals, plasma stearic acid, *trans* fatty acids intake, and breastfeeding duration and negatively associated with erythrocyte margaroleic acid. DHM cholecalciferol was associated with plasma cholecalciferol levels and dairy intake. Other weaker associations were found for free thiamine, free riboflavin, pyridoxal, dehydroascorbic acid, 25(OH)D_3_, total MUFAs, total PUFAs, and linoleic acid in DHM. We found no associations between the carbohydrates, proteins, lipid classes, phospholipids, and molecular species of triacyclglycerols in DHM and the clinical characteristics of the donors or their somatometric study, nutritional biochemical determinations, or dietary intake. 

Investigation of the composition of DHM and the factors that influence it is essential. Infants receiving DHM are in a state of nutritional emergency, and it is therefore desirable that the milk they receive is in accordance with their needs. Studies such as the present one may help to improve dietetic recommendations for milk donors to ensure a more suitable composition for DHM. However, much remains to be learned and it is possible that the development of personalized medicine will allow us to better understand the variability in the composition of DHM and the different needs of preterm infants. Answers to open questions are likely to be found in this new field. 

## Figures and Tables

**Table 1:** Donor characteristics (*n* = 114).

Characteristic	
Age (years)	35.6 (32.9, 38.7)
Weight (kg)	60.5 (55.2, 70.6)
Height (cm)	164.1 (6.5)
Pre-pregnancy BMI (kg/m^2^)	22.1 (20.6, 24.8)
Pre-pregnancy BMI (kg/m^2^) category	
Underweight (<18.5)	4 (3.5%)
Normal (18.5–24.9)	84 (73.7%)
Overweight (25–29.9)	15 (13.2%)
Obese (≥30)	11 (9.6%)
Current BMI (kg/m^2^)	22.9 (21.1, 25.1)
Current BMI (kg/m^2^) category	
Underweight (<18.5)	4 (3.5%)
Normal (18.5–24.9)	81 (71.1%)
Overweight (25–29.9)	16 (14.0%)
Obese (≥30)	13 (11.4%)
Gestational weight gain (kg)	11.3 (9.0, 14.0)
Postpartum weight retention (kg)	1.0 (−0.6, 2.5)
Number of living children	
0 ^a^–1	63 (55.3%)
2	39 (34.2%)
≥3	12 (10.5%)
Country of origin: Spain	102 (89.5%)
Education level	
Secondary studies	2 (1.8%)
Technical studies	14 (12.3%)
University studies	98 (86.0%)
Currently working	50 (43.9%)
Physical activity	
Sedentary	26 (22.8%)
Low activity	60 (52.6%)
Active/very active	28 (24.6%)
Tobacco consumption	
Previously	28 (24.6%)
Currently	1 (0.9%)
Passive smoking	24 (21.1%)
Active smoking	1 (0.9%)
Alcohol consumption	
Prior to pregnancy	55 (48.2%)
During pregnancy	1 (0.9%)
Currently	4 (3.5%)
Season during the study	
Spring	30 (26.3%)
Summer	16 (14.0%)
Autumn	42 (36.8%)
Winter	26 (22.8%)

Quantitative variables are expressed as means (standard deviations) when they were distributed parametrically and as medians (25th and 75th percentiles) when they were distributed non-parametrically. Qualitative variables are presented as the absolute and relative frequencies (%). ^a^ A fetal death at 22^+6^ weeks of gestational age and 450 g birthweight. Abbreviations: BMI, body mass index.

**Table 2:** Diseases, medication intake, and characteristics of the last pregnancy recorded for the donors (*n* = 114).

		*n* (%)
Diseases ^1^		41 (36.0%)
	Endocrinological and metabolic diseases *	10 (8.8%)
	Cardiovascular diseases (hypertension)	1 (0.9%)
	Respiratory diseases (asthma)	6 (5.3%)
	Immune diseases (allergy, psoriasis, atopy)	7 (6.1%)
	Spinal/medullary pathology	6 (5.3%)
	Miscellaneous **	13 (11.4%)
Medication intake ^1^		12 (10.5%)
	Oral contraceptives	4 (3.5%)
	Thyroid hormone replacement therapy	4 (3.5%)
	Other medicines ***	5 (4.4%)
Twin pregnancy		4 (3.5%)
Problems in the last pregnancy ^1^		36 (31.6%)
	Thyroid disorders	17 (14.9%)
	Preeclampsia	2 (1.8%)
	Gestational diabetes	2 (1.8%)
	Intrauterine fetal growth restriction	6 (5.3%)
	Intrauterine fetal death	3 (2.6%)
	Other problems ****	11 (9.6%)

^1^ The categories do not exclude each other. * Endocrinological and metabolic diseases included four cases of well-controlled hypothyroidism, four cases of subclinical thyroid disorders, and two cases of hypercholesterolemia. ** Miscellaneous included congenital amylase–sucrase deficiency, fatty liver, esophagitis, antiphospholipid syndrome, uveitis, migraines, polycystic ovary syndrome, cholesteatoma, venous insufficiency, recurrent urinary tract infections, human papillomavirus infection, and depression. *** Other medicines included antihistamines, antidepressants, proton-pump inhibitors, antivertiginous medications, and oral bronchodilators. **** Other problems included the threat of abortion, threat of premature birth, feto-fetal transfusion syndrome, chorioamnionitis, oligohydramnios, intrahepatic cholestasis, cytomegalovirus infection, bronchitis, and urinary tract infections.

**Table 3:** Characteristics of infants (*n* = 116).

Characteristic	n *	
Gestational age (weeks)	114	39^+4^ (38^+2^, 40^+2^); 22^+6^–42^+3^
Boy	116	55 (47.4%)
Birth weight (grams)	116	3195.0 (2795.0, 3472.5); 450.0–4640.0
Birth weight percentile ^1^		
≤25		32 (27.6%)
25–75	116	73 (62.9%)
≥75		11 (9.5%)
Age of infant (months)	114	
0–6		45 (39.5%)
6–12		43 (37.7%)
12–50		26 (22.8%)
Postmenstrual age of preterm infants (weeks)	19	50.3 (38.6, 78.2)
Weight percentile of breastfed child ^2^	113	
≤15		17 (15.0%)
15–85		77 (68.1%)
≥85		19 (16.8%)

Quantitative variables are expressed as medians (25th and 75th percentiles) because they were distributed non-parametrically. Ranges are shown after the semicolon. Qualitative variables are presented as the absolute and relative frequencies (%). * *n* = 116 includes a fetal death at 22^+6^ weeks of gestational age and two pairs of twins; *n* = 114 is due to the number of gestations; *n* = 113 excludes the fetal death, a twin baby who died in the first week of life, and one child with missing data. ^1^ Based on Olsen intrauterine growth curves [46]. ^2^ Based on the World Health Organization (WHO)’s child growth standards [47].

**Table 4:** Breastfeeding characteristics (*n* = 114).

Characteristic	*n*	*n* (%)
Donor previously	114	20 (17.5%)
Duration of lactation of the previous child (months)	51 ^a^	
0		1(2.0%)
3–6		2 (3.9%)
6–12		10 (19.6%)
12–24		22 (43.1%)
≥24		16 (31.4%)
Current lactation stage (months)	114	7.0 (4.8, 12.0); 1.8–50.0
Type of lactation	113 ^b^	
Exclusive		52 (46.0%)
Partial		61 (54.0%)
Sum of breastfeeding times plus daily milk pumping sessions	114	
<5		12 (10.5%)
5–10		66 (57.9%)
>10		33 (28.9%)
Missing data		3 (2.6%)
Tandem breastfeeding	114	5 (4.4%)
Breastfeeding twins	114	1 (0.9%)
Type of milk extraction *	114	
Manual		7 (6.1%)
Mechanical breast pump		12 (10.5%)
Simple electric breast pump		82 (71.9%)
Double electric breast pump		15 (13.2%)

Quantitative variables are expressed as medians (25th and 75th percentiles) because they were distributed non-parametrically. Ranges are shown after the semicolon. Qualitative variables are presented as the absolute and relative frequencies (%). ^a^ Number of donors with previous children. ^b^ Due to one fetal death. * The categories do not exclude each other.

**Table 5:** Macronutrient composition (g/100 mL milk), lipid classes’ profile (g/100 g fat), relative composition of phospholipids (g/100 g polar lipids), and molecular species of triacylglycerols content (g/100 g fat).

Nutrient	Donors	
	*n*	Mean (SE)
Macronutrients (g/100 mL milk)		
Lipids	103	3.13 (0.17)
Carbohydrates		7.73 (0.03)
Proteins		1.17 (0.03)
Lipid classes (g/100 g fat)		
Triacylglycerols	20	96.19 (93.87, 97.26)
Diacylglycerols		3.43 (2.47, 5.50)
Monoacylglycerols		0.03 (0.02, 0.07)
Free fatty acids + cholesterol		0.31 (0.22, 0.51)
Polar lipids		0.05 (0.01)
Phospholipids (g/100 g of polar lipids)		
Phosphatidylethanolamine	20	24.63 (7.88)
Phosphatidylcholine		30.95 (5.00)
Sphingomyelin		44.43 (11.09)
Triacylglycerols (g/100 g fat) *		
CN24	20	0.01 (0.01, 0.02)
CN26		0.10 (0.03)
CN28		0.07 (0.05, 0.12)
CN30		0.19 (0.14, 0.27)
CN32		0.26 (0.19, 0.41)
CN34		0.33 (0.13, 0.44)
CN36		0.36 (0.22, 0.65)
CN38		1.57 (0.68)
CN40		2.02 (0.54)
CN42		2.70 (0.91)
CN44		5.02 (1.37)
CN46		7.51 (1.50)
CN48		10.72 (1.43)
CN50		14.71 (2.16)
CN52		36.89 (4.83)
CN54		17.30 (5.10)

Variables are expressed as means (standard deviations) when they were distributed parametrically and as medians (25th and 75th percentiles) when they were distributed non-parametrically. * Molecular species of triacylglycerols according to their carbon number (CN).

**Table 6:** Fatty acid composition (g/100 g fat) of donor human milk.

Fatty Acid (%)	Common Name	Donors (*n* = 108)	Reference Values	
			European [52] ^1^	World [73] ^2^
Saturated Fatty Acids (SFAs)				
C6:0	Caproic	0.11 (0.02)	0.08 ± 0.02	0.13 ± 0.47
C8:0	Caprylic	0.18 (0.16, 0.21)	0.22 ± 0.06	0.21 ± 0.22
C10:0	Capric	1.20 (0.29)	1.44 ± 0.34	1.37 ± 0.86
C12:0	Lauric	5.42 (1.58)	5.46 ± 1.84	5.7 ± 2.81
C14:0	Myristic	5.88 (4.91, 7.75)	6.19 ± 1.93	6.56 ± 3.05
C15:0	Pentadecanoic	0.18 (0.13, 0.25)		
C15:0 ai	C15:0 anteiso	0.02 (0.02, 0.03)		
C15:0 i	C15:0 iso	0.03 (0.02, 0.05)		
C16:0 i	C16:0 iso	0.02 (0.01, 0.03)		
C16:0	Palmitic	19.61 (2.45)	21.94 ± 2.92	21.5 ± 4.82
C17:0 ai	C17:0 anteiso	0.04 (0.03, 0.06)		
C17:0 i	C17:0 iso	0.27 (0.06)		
C17:0	Margaric	0.19 (0.15, 0.23)		0.31 ± 0.15
C18:0	Stearic	5.72 (4.96, 6.49)	6.68 ± 1.59	6.36 ± 2.07
C20:0	Arachidic	0.16 (0.11, 0.21)	0.17 ± 0.04	0.23 ± 0.17
Monounsaturated Fatty Acids (MUFAs)				
C14:1 *cis*-9 (n5)	Myristoleic	0.07 (0.04, 0.11)		
C16:1 *cis*-9 (n7)	Palmitoleic	1.47 (1.19, 1.76)	2.21 ± 0.64	2.3 ± 0.92
C17:1	Margaroleic	0.07 (0.03)		
∑ C18:1 *trans*		0.22 (0.13, 0.34)	0.66 ± 0.35	
C18:1 *cis*-9 (n9)	Oleic	38.23 (4.98)	35.59 ± 4.17	32.6 ± 5.84
C18:1 *cis*-11 (n7)	Cis vaccenic	1.61 (0.28)	2.38 ± 0.53	
C20:1 (n9)	Gondoic	0.53 (0.36, 0.82)	0.38 ± 0.12	0.46 ± 0.28
*n*-6 Polyunsaturated Fatty Acids (*n*-6 PUFAs)				
C18:2 (n6)	Linoleic (LA)	14.79 (12.37, 17.19)	14.00 ± 4.95	15.7 ± 7.15
C20:2 (n6)	Eicosadienoic	0.25 (0.19, 0.35)	0.26 ± 0.07	0.37 ± 0.19
C20:3 (n6)	Dihomo-γ-linolenic	0.33 (0.23, 0.44)	0.31 ± 0.09	0.37 ± 0.18
C20:4 (n6)	Arachidonic (AA)	0.54 (0.18)	0.44 ± 0.12	0.50 ± 0.25
*n*-3 Polyunsaturated Fatty Acids (*n*-3 PUFAs)				
C18:3 (n3)	Linolenic (ALA)	0.50 (0.40, 0.62)	0.94 ± 0.55	1.11 ± 1.05
C22:5 (n3)	Docosapentaenoic (DPA)	0.07 (0.05, 0.11)		
C22:6 (n3)	Docosahexaenoic (DHA)	0.28 (0.17, 0.45)	0.34 ± 0.35	0.37 ± 0.31
*n*-7 Polyunsaturated Fatty Acids (*n*-7 PUFAs)				
C18:2 c9, t11 (n7)	Rumenic	0.08 (0.04, 0.12)		
Fatty Acid Families				
Not identified		0.20 (0.15, 0.25)		
SFAs		39.83 (37.0, 42.1)	42.23 ± 5.29	42.2 ± 7.73
MUFAs		42.49 (5.22)	41.34 ± 4.48	36.3 ± 6.46
PUFAs		16.71 (14.83, 19.37)	16.43 ± 5.07	21.2 ± 8.18
SCFAs		0.11 (0.10, 0.12)		
MCFAs (C8–C15)		13.02 (11.13, 16.18)		
LCFAs (C16–C18)		84.0 (80.71, 86.35)		
VLCFAs (C20–C24)		2.34 (2.02, 3.16)		
*n*-6 PUFAs		15.77 (13.46, 18.44)		17.8 ± 7.51
*n*-3 PUFAs		0.87 (0.72, 1.17)		1.88 ± 2.63
*n*-6 PUFAs/*n*-3 PUFAs		17.21 (13.31, 24.81)		
LA/ALA ratio		28.68 (22.52, 40.31)		
ARA/DHA ratio		1.83 (1.32, 3.06)	1.68 ± 0.89	

Variables are expressed as means (standard deviations) when they were distributed parametrically and as medians (25th and 75th percentiles) when they were distributed non-parametrically. ^1^ Data (mean ± standard deviation) from 223 lactating mothers at a breastfeeding stage of 120 ± 5 days. ^2^ Data (mean ± standard deviation) from mature milk. Abbreviations: SCFAs, short-chain fatty acids; MCFAs, medium-chain fatty acids; LCFAs, long-chain fatty acids; VLCFAs, very-long-chain fatty acids.

**Table 7:** Vitamin, mineral, and trace element composition of donor human milk (*n* = 113).

Nutrient ^1^	Donors		Mature Milk Nutrient Concentration Reference
	*n*	Concentration	
Free thiamin, B1 (UPLC-MS/MS) mcg/L	113	18.10 (10.03, 28.43)	Free thiamin 18.5 [74] Total thiamin 180 [75]
Free riboflavin, B2 (UPLC-MS/MS) mcg/L	113	47.30 (23.58, 99.90)	Free riboflavin 11.2 [74] Total riboflavin 364 [76]
Nicotinamide, B3 (UPLC-MS/MS) mcg/L	113	46.73 (28.50, 82.58)	Nicotinamide 275 [74] Total niacin 2100 [77]
Pantothenic acid, B5 (UPLC-MS/MS) mcg/L	113	2264.90 (1864.90, 2540.00)	2500 [78] 1304 [74]
Pyridoxal, B6 (UPLC-MS/MS) mcg/L	113	36.73 (27.80, 53.30)	Pyridoxal 96 [74] B6 130 [79]
Folic acid, B9 (UPLC-MS/MS) mcg/L	113	19.88 (7.02)	Folate 80 [79]
Cobalamin, B12 (competitive immunoassay)	113		
pM		490.63 (74.30)	
mcg/L		0.66 (0.10)	0.5 [80]
Ascorbic acid (HPLC-DAD) mg/dL	112	3.91 (1.71)	
Dehydroascorbic acid (HPLC-DAD) mg/dL	112	1.91 (1.29, 3.38)	
Vitamin C * (HPLC-DAD)	112		
mg/dL		6.37 (1.41)	
mg/L		63.70 (14.10)	35–90 [81]
Retinol (HPLC with fluorescence and UV detector)	112		
mcg/dL		41.15 (26.80, 72.48)	
mcg/L		411.50 (268.00, 724.80)	530 [82]
Vitamin D_3_ (UPLC–electrospray ionization/tandem MS)	112		
pg/mL		1603.65 (373.83, 5279.93)	
mcg/L		1.60 (0.37, 5.28)	0.25–2 [83]
25(OH)D_3_ (UPLC–electrospray ionization/tandem MS)	112		
pg/mL		53.90 (27.13, 109.85)	
mcg/L		0.05 (0.03, 0.11)	
α-tocopherol (HPLC with fluorescence and UV detector)	112		
mcg/dL		463.80 (373.33, 586.76)	
mg/L		4.64 (3.73, 5.87)	4.6 [84]
γ-tocopherol (HPLC with fluorescence and UV detector)	112		
mcg/dL		50.19 (36.43, 67.08)	
mg/L		0.50 (0.36, 0.67)	0.45 [74]
Vitamin E (as TE) **	112		
mcg/dL		488.02 (163.13)	
mg/L		4.88 (1.63)	5.2 [74]
Iodine (ICP-MS) ppb (mcg/L)	113	148.45 (98.95, 204.98)	50–100 [79] 100–200 [85,86]
Calcium (ICP-MS) ppm (mg/L)	113	99.10 (59.70, 127.35)	200–300 [87]
Phosphorous (ICP-MS) ppm (mg/L)	113	132.47 (114.00, 150.40)	120–140 [79,88]
Selenium (ICP-MS) ppb (mcg/L)	113	10.88 (9.30, 12.68)	18 [89]

Variables are expressed as means (standard deviations) when they were distributed parametrically and as medians (25th and 75th percentiles) when they were distributed non-parametrically. *n* is the number of donors with data for the nutrient under study. ^1^ The units of our results were converted to the units of the reference values for comparability. * Vitamin C = ascorbic acid + dehydroascorbic acid. ** Vitamin E = α-tocopherol (mg) + 0.25 × γ-tocopherol (mg) [90]. Abbreviations: UPLC, ultra-performance liquid chromatography; MS/MS, tandem mass spectrometry; HPLC, high-performance liquid chromatography; DAD, diode array detector; UV, ultraviolet; MS, mass spectrometry; TE, tocopherol equivalent; ICP, inductively coupled plasma; ppb, parts per billion; ppm, parts per million.

**Table 8:** Multivariate analysis of lipids in donor human milk.

Fatty Acids (% of Fat)	Associated Variables	Beta	SE	t	P > |t|	95% CI
Total SFAs ^a^ Observations = 105 R^2^ = 0.34	DMA in erythrocytes (% of fat in erythrocytes)	2.700	0.795	3.40	0.001	[1.122, 4.277]
	C17:1 in erythrocytes (% of fat in erythrocytes)	−13.556	3.565	−3.80	<0.001	[−20.629, −6.483]
	Plasma C18:0 (% of fat in plasma)	2.023	0.550	3.68	<0.001	[0.932, 3.114]
	*Trans* fatty acids (average % kcals. consumed from *trans* fatty acids per day from the dietary record)	5.789	2.171	2.67	0.009	[1.481, 10.097]
	Breastfeeding time (months)	0.203	0.071	2.86	0.005	[0.062, 0.344]
Total MUFAs ^b^ Observations = 103 R^2^ = 0.16	Plasma MUFAs (% of fat in plasma)	0.458	0.165	2.77	0.007	[0.130, 0.786]
	C17:1 in erythrocytes (% of fat in erythrocytes)	13.523	4.895	2.76	0.007	[3.810, 23.235]
Total PUFAs Observations = 108 R^2^ = 0.04	PUFAs (average intake in g/day from the dietary record)	0.139	0.062	2.23	0.028	[0.015, 0.263]
Linoleic acid ^c^ Observations = 106 R^2^ = 0.10	Linoleic acid in erythrocytes (% of fat in erythrocytes)	0.633	0.209	3.02	0.003	[0.217, 1.048]
	Meat, fish, eggs (average servings/day from the dietary record)	0.684	0.281	2.44	0.016	[0.128, 1.241]
DHA ^d^ Observations = 104 R^2^ = 0.45	Plasma DHA (% of fat in plasma)	0.164	0.334	4.91	<0.001	[0.098, 0.230]
	DHA (average intake in g/day from the dietary record)	0.382	0.075	5.12	<0.001	[0.234, 0.530]
Total omega-3 ^e^ Observations = 106 R^2^ = 0.29	DHA (average intake in g/day from the dietary record)	0.783	0.120	6.51	<0.001	[0.544, 1.021]

^a^ After a diagnosis of the model, the data entries 2 and 32 were removed. ^b^ After a diagnosis of the model, the data entries 2, 32, and 71 were removed. ^c^ After a diagnosis of the model, the data entries 19 and 135 were removed. ^d^ After diagnosis of the model, the data entries 6, 68, and 73 were removed. ^e^ After a diagnosis of the model, the data entries 6 and 68 were removed. Abbreviations: SE, standard error; SFAs, saturated fatty acids; DMA, dimethyl acetals, MUFAs, monounsaturated fatty acids; PUFAs, polyunsaturated fatty acids; DHA, docosahexaenoic acid.

## Data Availability

The data presented in this study are available on request from the corresponding author. The data are not publicly available due to privacy issues.

## Supplementary Materials

The following supporting information can be downloaded at: https://www.mdpi.com/article/10.3390/nu15153486/s1, Table S1: Consumption of the pharmacological supplements during pregnancy and the lactation of the human milk donors (*n* = 113); Table S2: Diet survey: five-day dietary record. Daily nutrient intake of the human milk donors; Table S3: Prevalence of inadequate intake of specific nutrients in human milk donors; Table S4: Diet survey: five-day dietary diaries. Number of food servings per day consumed by the participants, healthy eating index (HEI), and records of supplement and iodized salt intake among the human milk donors; Table S5: Diet survey: food frequency questionnaire (FFQ, the number of food servings per day or week consumed by the participants) results for the human milk donors; Table S6: Erythrocyte and plasma fatty acid compositions (g/100 g of total FAs) for the human milk donors; Table S7: Erythrocyte, plasma, and urine concentrations of nutrients and biochemical determinations for the human milk donors.

## References

[1] Arslanoglu S., Corpeleijn W., Moro G., Braegger C., Campoy C., Colomb V., Decsi T., Domellöf M., Fewtrell M., Hojsak I. (2013). Donor human milk for preterm infants: Current evidence and research directions. J. Pediatr. Gastroenterol. Nutr..

[2] Moro G.E., Billeaud C., Rachel B., Calvo J., Cavallarin L., Christen L., Escuder-Vieco D., Gaya A., Lembo D., Wesolowska A. (2019). Processing of Donor Human Milk: Update and Recommendations From the European Milk Bank Association (EMBA). Front. Pediatr..

[3] Weaver G., Bertino E., Gebauer C., Grovslien A., Mileusnic-Milenovic R., Arslanoglu S., Barnett D., Boquien C.Y., Buffin R., Gaya A. (2019). Recommendations for the establishment and operation of Human Milk Banks in Europe: A consensus statement from the European Milk Bank Association (EMBA). Front. Pediatr..

[4] Abrams S.A., Landers S., Noble L.M., Poindexter B.B., AAP Comittee on Nutrition, AAP Section on Breastfeeding, AAP Comittee on Fetus and Newborn (2017). Donor human milk for the high-risk infant: Preparation, safety, and usage options in the United States. Pediatrics.

[5] Martini S., Beghetti I., Annunziata M., Aceti A., Galletti S., Ragni L., Donti A., Corvaglia L. (2021). Enteral Nutrition in Term Infants with Congenital Heart Disease: Knowledge Gaps and Future Directions to Improve Clinical Practice. Nutrients.

[6] National Academies of Sciences, Engineering and Medicine, Health and Medicine Division, Food and Nutrition Board (2020). Nutrition During Pregnancy and Lactation: Exploring New Evidence: Proceedings of a Workshop.

[7] Dror D.K., Allen L.H. (2018). Overview of nutrients in human milk. Adv. Nutr..

[8] Bravi F., Wiens F., Decarli A., Dal Pont A., Agostoni C., Ferraroni M. (2016). Impact of maternal nutrition on breast-milk composition: A systematic review. Am. J. Clin. Nutr..

[9] Keikha M., Bahreynian M., Saleki M., Kelishadi R. (2017). Macro- and Micronutrients of Human Milk Composition: Are They Related to Maternal Diet? A Comprehensive Systematic Review. Breastfeed. Med..

[10] Allen L.H. (2012). B Vitamins in Breast Milk: Relative Importance of Maternal Status and Intake, and Effects on Infant Status and Function. Adv. Nutr. An Int. Rev. J..

[11] Smilowitz J.T., Allen L.H., Dallas D.C., McManaman J., Raiten D.J., Rozga M., Sela D.A., Seppo A., Williams J.E., Young B.E. (2023). Ecologies, synergies, and biological systems shaping human milk composition—A report from “Breastmilk Ecology: Genesis of Infant Nutrition (BEGIN)” Working Group 2. Am. J. Clin. Nutr..

[12] Allen L.H. (2005). Multiple micronutrients in pregnancy and lactation: An overview. Am. J. Clin. Nutr..

[13] Lima M.S.R., Dimenstein R., Ribeiro K.D.S. (2014). Vitamin e concentration in human milk and associated factors: A literature review. J. Pediatr..

[14] Barrera C., Valenzuela R., Chamorro R., Bascuñán K., Sandoval J., Sabag N., Valenzuela F., Valencia M.P., Puigrredon C., Valenzuela A. (2018). The impact of maternal diet during pregnancy and lactation on the fatty acid composition of erythrocytes and breast milk of chilean women. Nutrients.

[15] Bu T., Tang D., Liu Y., Chen D. (2021). Trends in Dietary Patterns and Diet-related Behaviors in China. Am. J. Health Behav..

[16] Grech A., Rangan A., Allman-Farinelli M. (2018). Macronutrient Composition of the Australian Population’s Diet; Trends from Three National Nutrition Surveys 1983, 1995 and 2012. Nutrients.

[17] Fulgoni K., Fulgoni V.L. (2021). Trends in total, added, and natural phosphorus intake in adult americans, nhanes 1988–1994 to nhanes 2015–2016. Nutrients.

[18] Christian P., Smith E.R., Lee S.E., Vargas A.J., Bremer A.A., Raiten D.J. (2021). The need to study human milk as a biological system. Am. J. Clin. Nutr..

[19] Hampel D., Shahab-Ferdows S., Islam M.M., Peerson J.M., Allen L.H. (2017). Vitamin Concentrations in Human Milk Vary with Time within Feed, Circadian Rhythm, and Single-Dose Supplementation. J. Nutr..

[20] Dawodu A., Tsang R.C. (2012). Maternal Vitamin D Status: Effect on Milk Vitamin D Content and Vitamin D Status of Breastfeeding Infants. Adv. Nutr..

[21] Ortega R.M., López-Sobaler A.M., Martínez R.M., Andrés P., Elena Quintas M. (1998). Influence of smoking on vitamin E status during the third trimester of pregnancy and on breast-milk tocopherol concentrations in Spanish women. Am. J. Clin. Nutr..

[22] Perrin M.T., Belfort M.B., Hagadorn J.I., McGrath J.M., Taylor S.N., Tosi L.M., Brownell E.A. (2020). The nutritional composition and energy content of donor human milk: A systematic review. Adv. Nutr..

[23] Sierra-Colomina G., García-Lara N.R., Escuder-Vieco D., Alonso-Díaz C., Andrés Esteban E.M., Pallás-Alonso C.R. (2014). Donor milk volume and characteristics of donors and their children. Early Hum. Dev..

[24] Osbaldiston R., Mingle L.A. (2007). Characterization of human milk donors. J. Hum. Lact..

[25] Parker M.G., Stellwagen L.M., Noble L., Kim J.H., Poindexter B.B., Puopolo K.M., Section on Breastfeeding, Committee on Nutrition, Committee on Fetus and Newborn (2021). Promoting Human Milk and Breastfeeding for the Very Low Birth Weight Infant. Pediatrics.

[26] Peila C., Moro G.E., Bertino E., Cavallarin L., Giribaldi M., Giuliani F., Cresi F., Coscia A. (2016). The effect of holder pasteurization on nutrients and biologically-active components in donor human milk: A review. Nutrients.

[27] Leaf A., Lansdowne Z. (2014). Vitamins—Conventional uses and new insights. World Rev. Nutr. Diet..

[28] Van Zoeren-Grobben D., Schrijver J., Van den Berg H., Berger H.M. (1987). Human milk vitamin content after pasteurisation, storage, or tube feeding. Arch. Dis. Child..

[29] Romeu-Nadal M., Castellote A.I., López-Sabater M.C. (2008). Effect of cold storage on vitamins C and E and fatty acids in human milk. Food Chem..

[30] Nessel I., Khashu M., Dyall S.C. (2019). The effects of storage conditions on long-chain polyunsaturated fatty acids, lipid mediators, and antioxidants in donor human milk—A review. Prostaglandins Leukot. Essent. Fat. Acids.

[31] Wei W., Cheng J., Yang J., Chen C., Jin Q., Song J., Wang X. (2021). Phospholipid composition and fat globule structure change during low temperature storage of human milk. LWT.

[32] O’Connor D.L., Ewaschuk J.B., Unger S. (2015). Human milk pasteurization: Benefits and risks. Curr. Opin. Clin. Nutr. Metab. Care.

[33] Escuder-Vieco D., Rodríguez J.M., Espinosa-Martos I., Corzo N., Montilla A., García-Serrano A., Calvo M.V., Fontecha J., Serrano J., Fernández L. (2021). High-Temperature Short-Time and Holder Pasteurization of Donor Milk: Impact on Milk Composition. Life.

[34] Valentine C.J., Morrow G., Pennell M., Morrow A.L., Hodge A., Haban-Bartz A., Collins K., Rogers L.K. (2013). Randomized Controlled Trial of Docosahexaenoic Acid Supplementation in Midwestern U.S. Human Milk Donors. Breastfeed. Med..

[35] Ureta-Velasco N., Keller K., Escuder-Vieco D., Serrano J.C.E., García-Lara N.R., Pallás-Alonso C.R. (2022). Assessment of Iodine Concentration in Human Milk from Donors: Implications for Preterm Infants. Nutrients.

[36] Ureta-Velasco N., Keller K., Escuder-Vieco D., Fontecha J., Calvo M.V., Megino-Tello J., Serrano J.C.E., Romero Ferreiro C., García-Lara N.R., Pallás-Alonso C.R. (2023). Human Milk Composition and Nutritional Status of Omnivore Human Milk Donors Compared with Vegetarian/Vegan Lactating Mothers. Nutrients.

[37] Faul F., Erdfelder E., Lang A.G., Buchner A. (2007). G*Power 3: A Flexible Statistical Power Analysis Program for the Social, Behavioral, and Biomedical Sciences. Behav. Res. Methods.

[38] Ortega R., López-Sobaler A., Andrés P., Requejo A., Aparicio A., Molinero L. (2013). DIAL Software for Assessing Diets and Food Calculations.

[39] Institute of Medicine (1997). Dietary Reference Intakes for Calcium, Phosphorus, Magnesium, Vitamin D, and Fluoride.

[40] Institute of Medicine (1998). Dietary Reference Intakes for Thiamin, Riboflavin, Niacin, Vitamin B6, Folate, Vitamin B12, Pantothenic Acid, Biotin, and Choline.

[41] Institute of Medicine (2000). Dietary Reference Intakes for Vitamin C, Vitamin E, Selenium, and Carotenoids.

[42] Institute of Medicine (2001). Dietary Reference Intakes for Vitamin A, Vitamin K, Arsenic, Boron, Chromium, Copper, Iodine, Iron, Manganese, Molybdenum, Nickel, Silicon, Vanadium, and Zinc.

[43] Institute of Medicine (2005). Dietary Reference Intakes for Energy, Carbohydrate, Fiber, Fat, Fatty Acids, Cholesterol, Protein, and Amino Acids.

[44] Ross C.A., Taylor C.L., Yaktine A.L., Del Valle H.B., Institute of Medicine, Institute of Medicine (2011). Dietary Reference Intakes for Calcium and Vitamin D.

[45] EFSA (European Food Safety Authority) (2017). Dietary Reference Values for nutrients. Summ. Rep. EFSA Support. Publ..

[46] Olsen I.E., Groveman S.A., Lawson M.L., Clark R.H., Zemel B.S. (2010). New intrauterine growth curves based on United States data. Pediatrics.

[47] World Health Organization (2006). WHO Child Growth Standards: Length/Height-for-Age, Weight-for-Age, Weight-for-Length, Weight-for-Height and Body Mass Index-for-Age: Methods and Development.

[48] Allen L.H., Carriquiry A.L., Murphy S.P. (2020). Perspective: Proposed Harmonized Nutrient Reference Values for Populations. Adv Nutr.

[49] Ortega Anta R., Requejo Marcos A. (2015). Nutriguía. Manual de Nutrición Clínica.

[50] Kennedy E.T., Ohls J., Carlson S., Fleming K. (1995). The Healthy Eating Index: Design and applications. J. Am. Diet. Assoc..

[51] Dapcich V., Salvador Castell G., Ribas Barba L., Pérez Rodrigo C., Aranceta-Bartrina J., Serra Majem L., Sociedad Española de Nutrición Comunitaria (2004). Embarazo y lactancia. Necesidades especiales. Guía de la Alimentación Saludable.

[52] Giuffrida F., Fleith M., Goyer A., Samuel T.M., Elmelegy-Masserey I., Fontannaz P., Cruz-Hernandez C., Thakkar S.K., Monnard C., De Castro C.A. (2022). Human milk fatty acid composition and its association with maternal blood and adipose tissue fatty acid content in a cohort of women from Europe. Eur. J. Nutr..

[53] Graham J., Peerson J., Haskell M., Shrestha R., Brown K., Allen L. (2005). Erythrocyte riboflavin for the detection of riboflavin deficiency in pregnant Nepali women. Clin. Chem..

[54] Berger M.M., Shenkin A., Schweinlin A., Amrein K., Augsburger M., Biesalski H.K., Bischoff S.C., Casaer M.P., Gundogan K., Lepp H.L. (2022). ESPEN micronutrient guideline. Clin. Nutr..

[55] Ehsanian R., Anderson S., Schneider B., Kennedy D., Mansourian V. (2020). Prevalence of low plasma vitamin B1 in the stroke population admitted to acute inpatient rehabilitation. Nutrients.

[56] Petteys B.J., Frank E.L. (2011). Rapid determination of vitamin B2 (riboflavin) in plasma by HPLC. Clin. Chim. Acta.

[57] Andraos S., Jones B., Wall C., Thorstensen E., Kussmann M., Smith D.C., Lange K., Clifford S., Saffery R., Burgner D. (2021). Plasma B vitamers: Population epidemiology and parent-child concordance in children and adults. Nutrients.

[58] Allen L.H., Miller J.W., De Groot L., Rosenberg I.H., Smith A.D., Refsum H., Raiten D.J. (2018). Biomarkers of Nutrition for Development (BOND): Vitamin B-12 Review. J. Nutr..

[59] Pawlak R., Parrott S.J., Raj S., Cullum-Dugan D., Lucus D. (2013). How prevalent is vitamin B12 deficiency among vegetarians?. Nutr. Rev..

[60] Sobczyńska-Malefora A., Harrington D.J. (2018). Laboratory assessment of folate (Vitamin B9) status. J. Clin. Pathol..

[61] Bailey L.B., Stover P.J., McNulty H., Fenech M.F., Gregory J.F., Mills J.L., Pfeiffer C.M., Fazili Z., Zhang M., Ueland P.M. (2015). Biomarkers of nutrition for development-Folate review. J. Nutr..

[62] De Pee S., Dary O. (2002). Biochemical indicators of vitamin A deficiency: Serum retinol and serum retinol binding protein. J. Nutr..

[63] World Health Organization (2011). Serum Retinol Concentrations for Determining the Prevalence of Vitamin A Deficiency in Populations.

[64] U.S. Centers for Disease Control and Prevention (2012). Second National Report on Biochemical Indicators of Diet and Nutrition in the U.S. Population.

[65] Dietary Guidelines Advisory Committee (2015). Scientific Report of the 2015 Dietary Guidelines Advisory Committee: Advisory Report to the Secretary of Health and Human Services and the Secretary of Agriculture.

[66] Dror D.K., Allen L.H. (2011). Vitamin E deficiency in developing countries. Food Nutr. Bull..

[67] Henjum S., Manger M., Hampel D., Brantsæter A.L., Shahab-Ferdows S., Bastani N.E., Strand T.A., Refsum H., Allen L.H. (2020). Vitamin B12 concentrations in milk from Norwegian women during the six first months of lactation. Eur. J. Clin. Nutr..

[68] Norman E.J., Morrison J.A. (1993). Screening elderly populations for cobalamin (vitamin B12) deficiency using the urinary methylmalonic acid assay by gas chromatography mass spectrometry. Am. J. Med..

[69] World Health Organization Urinary Iodine Concentrations for Determining Iodine Status Deficiency in Populations. Vitamin and Mineral Nutrition Information System.

[70] Ahn J., Lee J.H., Lee J., Baek J.Y., Song E., Oh H.S., Kim M., Park S., Jeon M.J., Kim T.Y. (2020). Association between urinary sodium levels and iodine status in Korea. Korean J. Intern. Med..

[71] Foley K.F., Boccuzzi L. (2010). Urine calcium: Laboratory measurement and clinical utility. Lab. Med..

[72] Fernández-Ruiz L., Rodelo Haad C., Rodríguez-Portillo M., Santamaría-Olmo R. (2020). Variation of phosphaturia according to phosphorus intake. Actual. Medica.

[73] Zhang Z., Wang Y., Yang X., Cheng Y., Zhang H., Xu X., Zhou J., Chen H., Su M., Yang Y. (2022). Human Milk Lipid Profiles around the World: A Systematic Review and Meta-Analysis. Adv. Nutr..

[74] Gibson R.S., Rahmannia S., Diana A., Leong C., Haszard J.J., Hampel D., Reid M., Erhardt J., Suryanto A.H., Sofiah W.N. (2020). Association of maternal diet, micronutrient status, and milk volume with milk micronutrient concentrations in Indonesian mothers at 2 and 5 months postpartum. Am. J. Clin. Nutr..

[75] Turck D., Bresson J.L., Burlingame B., Dean T., Fairweather-Tait S., Heinonen M., Hirsch-Ernst K.I., Mangelsdorf I., McArdle H.J., EFSA Panel on Dietetic Products, Nutrition and Allergies (NDA) (2016). Dietary reference values for thiamin. EFSA J..

[76] Turck D., Bresson J.-L., Burlingame B., Dean T., Fairweather-Tait S., Heinonen M., Hirsch-Ernst K., Mangelsdorf I., McArdle H., EFSA Panel on Dietetic Products, Nutrition and Allergies (NDA) (2017). Dietary Reference Values for riboflavin. EFSA J..

[77] EFSA Panel on Dietetic Products, Nutrition and Allergies (NDA) (2014). Scientific Opinion on Dietary Reference Values for niacin. EFSA J..

[78] EFSA Panel on Dietetic Products, Nutrition and Allergies (NDA) (2014). Scientific Opinion on Dietary Reference Values for pantothenic acid. EFSA J..

[79] EFSA Panel on Dietetic Products, Nutrition and Allergies (NDA) (2013). Scientific Opinion on nutrient requirements and dietary intakes of infants and young children in the European Union. EFSA J..

[80] EFSA Panel on Dietetic Products, Nutrition and Allergies (NDA) (2015). Scientific Opinion on Dietary Reference Values for cobalamin (vitamin B12). EFSA J..

[81] EFSA Panel on Dietetic Products, Nutrition and Allergies (NDA) (2013). Scientific Opinion on Dietary Reference Values for vitamin C. EFSA J..

[82] EFSA Panel on Dietetic Products, Nutrition and Allergies (NDA) (2015). Scientific Opinion on Dietary Reference Values for vitamin A. EFSA J..

[83] EFSA Panel on Dietetic Products, Nutrition and Allergies (NDA) (2016). Scientific opinion on dietary reference values for vitamin D. EFSA J..

[84] EFSA Panel on Dietetic Products, Nutrition and Allergies (NDA) (2015). Scientific Opinion on Dietary Reference Values for vitamin E as α-tocopherol. EFSA J..

[85] Semba R.D., Delange F. (2001). Iodine in human milk: Perspectives for infant health. Nutr. Rev..

[86] Andersson M., Braegger C.P. (2022). The role of iodine for thyroid function in lactating women and infants. Endocr. Rev..

[87] EFSA Panel on Dietetic Products, Nutrition and Allergies (NDA) (2015). Scientific Opinion on Dietary Reference Values for calcium. EFSA J..

[88] EFSA Panel on Dietetic Products, Nutrition and Allergies (NDA) (2015). Scientific Opinion on Dietary Reference Values for phosphorus. EFSA J..

[89] EFSA Panel on Dietetic Products, Nutrition and Allergies (NDA) (2014). Scientific Opinion on Dietary Reference Values for selenium. EFSA J..

[90] LASER Analytica (2014). Comprehensive literature search and review of breast milk composition as preparatory work for the setting of dietary reference values for vitamins and minerals. EFSA Support. Publ..

[91] Castillo F., Castillo-Ferrer F.J., Cordobilla B., Domingo J.C. (2021). Inadequate content of docosahexaenoic acid (DHA) of donor human milk for feeding preterm infants: A comparison with mother’s own milk at different stages of lactation. Nutrients.

[92] Castillo Salinas F., Montaner Ramón A., Castillo Ferrer F.-J., Domingo-Carnice A., Cordobilla B., Domingo J.C. (2022). Erythrocyte Membrane Docosahexaenoic Acid (DHA) and Lipid Profile in Preterm Infants at Birth and Over the First Month of Life: A Comparative Study with Infants at Term. Nutrients.

[93] Makrides M., Neumann M., Gibson R. (1996). Effect of maternal docosahexaenoic (DHA) supplementation on breast milk composition. Eur. J. Clin. Nutr..

[94] Valentine C.J., Morrow G., Fernandez S., Gulati P., Bartholomew D., Long D., Welty S.E., Morrow A.L., Rogers L.K. (2010). Docosahexaenoic acid and amino acid contents in pasteurized donor milk are low for preterm infants. J. Pediatr..

[95] Baack M.L., Norris A.W., Yao J., Colaizy T. (2012). Long Chain Polyunsaturated Fatty Acid Levels in U.S. Donor Human Milk: Meeting the Needs of Premature Infants?. J. Perinatol..

[96] Francois C.A., Connor S.L., Bolewicz L.C., Connor W.E. (2003). Supplementing lactating women with flaxseed oil does not increase docosahexaenoic acid in their milk. Am. J. Clin. Nutr..

[97] Valentine C.J. (2012). Maternal Dietary DHA Supplementation to Improve Inflammatory Outcomes in the Preterm Infant. Adv. Nutr..

[98] García-Maldonado E., Alcorta A., Zapatera B., Vaquero M.P. (2023). Changes in fatty acid levels after consumption of a novel docosahexaenoic supplement from algae: A crossover randomized controlled trial in omnivorous, lacto-ovo vegetarians and vegans. Eur. J. Nutr..

[99] Perrin M.T., Pawlak R., Dean L.L., Christis A., Friend L. (2019). A cross-sectional study of fatty acids and brain-derived neurotrophic factor (BDNF) in human milk from lactating women following vegan, vegetarian, and omnivore diets. Eur. J. Clin. Nutr..

[100] Scopesi F., Ciangherotti S., Lantieri P.B., Risso D., Bertini I., Campone F., Pedrotti A., Bonacci W., Serra G. (2001). Maternal dietary PUFAs intake and human milk content relationships during the first month of lactation. Clin. Nutr..

[101] Kodentsova V.M., Vrzhesinskaya O.A. (2006). Evaluation of the vitamin status in nursing women by vitamin content in breast milk. Bull. Exp. Biol. Med..

[102] Dror D.K., Allen L.H. (2018). Vitamin B-12 in humanmilk: A systematic review. Adv. Nutr..

[103] Salmenperä L. (1984). Vitamin C nutrition during prolonged lactation: Optimal in infants while marginal in some mothers. Am. J. Clin. Nutr..

[104] Hoppu U., Rinne M., Salo-Väänänen P., Lampi A.-M., Piironen V., Isolauri E. (2005). Vitamin C in breast milk may reduce the risk of atopy in the infant. Eur. J. Clin. Nutr..

[105] Dorea J.G. (2002). Selenium and breast-feeding. Br. J. Nutr..

[106] Miller E.M., Aiello M.O., Fujita M., Hinde K., Milligan L., Quinn E.A. (2013). Field and laboratory methods in human milk research. Am. J. Hum. Biol..

[107] Institute of Medicine (US) Committee on Nutritional Status during Pregnancy and Lactation (1991). Nutrition during Lactation.

