# Supplementary material for: Associations of Dietary Intake and Nutrient Status with Micronutrient and Lipid Composition in Breast Milk of Donor Women
Source: Nutrients. 2023 Aug 7;15(15):3486. doi: 10.3390/nu15153486 (PMC10421487; doi:10.3390/nu15153486)
Supplement: Supplementary file 1 [file nutrients-15-03486-s001.zip › nutrients-2488822-supplementary.pdf]

Supplementary Data Article Associations of Dietary Intake and Nutrient Status with Micronutrient and Lipid Composition in Breast Milk of Donor Women

Table S1. Consumption of the pharmacological supplements during pregnancy and the lactation of the human milk donors (*n* = 113)

Pharmacological Supplement	*n* (%)	Daily Dose
Vitamin A, mcg		
Pregnancy	17 (10.0)	700.0 (400.0, 700.0)
Lactation	58 (51.3)	
Previously	14 (12.4)	800.0 (800.0, 800.0)
Currently	44 (38.9)	800.0 (400.0, 800.0)
Vitamin D, mcg		
Pregnancy	61 (54.0)	10.0 (5.0, 10.0)
Lactation	66 (58.4)	
Previously	15 (13.3)	5.0 (5.0, 5.0)
Currently	51 (45.1)	5.0 (3.5, 5.0)
Vitamin E, mg		
Pregnancy	30 (26.5)	12.0 (12.0, 12.0)
Lactation	63 (55.8)	
Previously	15 (13.3)	12.0 (12.0, 12.0)
Currently	48 (42.5)	12.0 (8.4, 12.0)
Vitamin C, mg		
Pregnancy	59 (52.2)	60.0 (40.0, 80.0)
Lactation	65 (57.5)	
Previously	16 (14.2)	80.0 (80.0, 80.0)
Currently	49 (43.4)	80.0 (60.0, 80.0)
Vitamin B1, thiamine, mg		
Pregnancy	59 (52.2)	1.1 (1.1, 1.1)
Lactation	64 (56.6)	
Previously	16 (14.2)	1.1 (1.1, 1.1)
Currently	48 (42.5)	1.1 (0.7, 1.1)
Vitamin B2, riboflavin, mg		
Pregnancy	59 (52.2)	1.4 (1.4, 1.4)
Lactation	64 (56.6)	
Previously	16 (14.2)	1.4, (1.4, 1.4)
Currently	48 (42.5)	1.4 (0.8, 1.4)
Vitamin B3, niacin, mg		
Pregnancy	59 (52.2)	16.0 (16.0, 16.0)
Lactation	64 (56.6)	
Previously	16 (14.2)	16.0 (16.0, 16.0)
Currently	48 (42.5)	16.0 (8.0, 16.0)
Vitamin B5, pantothenic, mg		
Pregnancy	59 (52.2)	6.0 (6.0, 6.0)
Lactation	64 (56.6)	
Previously	16 (14.2)	6.0 (6.0, 6.0)
Currently	48 (42.5)	6.0 (3.0, 6.0)
Vitamin B6, pyridoxine, mg		
Pregnancy	60 (53.1)	1.4 (1.4, 1.4)
Lactation	64 (56.6)	
Previously	16 (14.2)	1.4 (1.4, 1.4)

Currently	48 (42.5)	1.4 (0.9, 1.4)
Vitamin B7, biotin, mcg		
Pregnancy	59 (52.2)	50.0 (50.0, 50.0)
Lactation	64 (56.6)	
Previously	16 (14.2)	50.0 (50.0, 50.0)
Currently	48 (42.5)	50.0 (29.0, 50.0)
Vitamin B9, folic acid, mcg		
Pregnancy	109 (96.5)	400.0 (400.0, 400.0)
Lactation	94 (83.2)	
Previously	22 (19.5)	400.0 (200.0, 400.0)
Currently	72 (63.7)	300.0 (200.0, 400.0)
Vitamin B12, cobalamin, mcg		
Pregnancy	106 (93.8)	2.3 (2.0, 2.5)
Lactation	93 (82.3)	
Previously	22 (19.5)	2.5 (2.0, 2.5)
Currently	71 (2.8)	2.0 (2.0, 2.5)
Iodine, mcg		
Pregnancy	108 (95.6)	200.00 (200.0, 200.0)
Lactation	97 (85.8)	
Previously	21 (18.6)	200.0 (200.0, 200.0)
Currently	76 (67.3)	200.0 (200.0, 200.0)
Calcium, mg		
Pregnancy	5 (4.4)	150.00 (24.0, 20.00)
Lactation	47 (41.6)	
Previously	12 (10.6)	200.0 (100.0, 200.0)
Currently	35 (31.0)	200.0 (100.0, 200.0)
Iron, mg		
Pregnancy	82 (72.6)	28.0 (28.0, 80.0)
Lactation	81 (71.7)	
Previously	27 (23.9)	15.0 (14.0, 80.0)
Currently	54 (47.8)	14.0 (14.0, 33.0)
Zinc, mg		
Pregnancy	54 (47.8)	10.0 (10.0, 10.0)
Lactation	61 (54.0)	
Previously	16 (14.2)	10.0 (10.0, 10.0)
Currently	45 (39.8)	10.0 (6.0, 10.0)
Selenium, mcg		
Pregnancy	51 (45.1)	55.0 (55.0, 55.0)
Lactation	59 (52.2)	
Previously	15 (13.3)	20.0 (20.0, 30.0)
Currently	44 (38.9)	20.0 (10.0, 55.0)
Omega 3, g		
Pregnancy	59 (52.2)	0.20 (0.20, 0.22)
Lactation	61 (54.0)	
Previously	15 (13.3)	0.24 (0.16, 0.24)
Currently	46 (40.7)	0.24 (0.20, 0.24)
DHA, g		
Lactation	61 (54.0)	
Previously	15 (13.3)	0.20 (0.20,0.20)
Currently	46 (40.7)	0.20 (0.16, 0.24)

EPA, g		
Lactation	61 (54.0)	
Previously	15 (13.3)	0.04 (0.02, 0.04)
Currently	46 (40.7)	0.04 (0.02, 0.04)

The quantitative variables are presented as medians (25th, 75th percentiles) because they all followed a nonparametric distribution. The qualitative variables are expressed as the absolute and relative frequencies (%). Women who took supplements during lactation but had stopped taking them at the time of the study were classified in the "Previously" group. Women who were still taking supplements at the time of the study were classified in the "Currently" group. Abbreviations: DHA, docosahexaenoic acid; and EPA, eicosapentaenoic acid.

Table S2. Diet survey: five-day dietary record. Daily nutrients intake of the human milk donors

	Donors (*n* = 113)	Recommendations [a]	
		EFSA (PRI/AI *)	IOM (RDA/AI *)
Energy (Kcal)	2305.20 (2031.20, 2499.20)	[b]	
Protein (g)	93.74 (82.78, 103.92)	[c]	71
Total fat (g)	101.80 (86.10, 118.40)		
Saturated fat (g)	32.20 (27.42, 37.92)	ALAP	ALAP
Polyunsaturated fat (g)	15.26 (12.20, 19.02)		
Monounsaturated fat (g)	42.48 (35.74, 50.00)		
PUFAs/SFAs	0.52 (0.41, 0.66)		
(PUFAs + MUFAs)/SFAs	1.88 (1.64, 2.24)		
Kcal from carbohydrate (%)	44.13 (5.86)	45–60 **	45–65 **
Kcal from protein (%)	16.26 (15.04, 17.74)		10–35 **
Kcal from fat (%)	39.08 (5.92)	20–35 **	20–35 **
Kcal from trans fatty acids (%)	0.44 (0.29, 0.52)	ALAP	ALAP
Kcal from saturated fat (%)	12.56 (11.34, 14.06)		
Kcal from polyunsaturated fat (%)	6.06 (5.20, 7.48)		
Kcal from monounsaturated fat (%)	16.08 (14.53, 19.02)		
Kcal from *n*-3 fatty acids (%)	0.78 (0.65, 1.01)	0.5	0.6–1.2 **
n-6 fatty acids (g)	4.73 (2.09, 15.95)		13 *
n-3 fatty acids (g)	1.50 (0.43, 6.20)		1.3 *
n-6/*n* 3 fatty acids	7.11 (5.78, 8.82)		
Myristic acid C14:0 (g)	2.70 (1.98, 3.56)		
Palmitic acid C16:0 (g)	16.20 (13.02, 19.02)		
Palmitoleic acid C16:1 n7 (g)	1.52 (0.54)		
Stearic acid C18:0 (g)	6.96 (5.56, 8.26)		
Oleic acid C18:1n9c (g)	39.40 (32.30, 46.46)		
Linoleic acid C18:2n6c (g)	12.38 (9.58, 16.46)		
Linolenic acid C18:3n3 (g)	1.44 (1.13, 1.88)		
Eicosapentaenoic acid C20:5n3 (g)	0.08 (0.04, 0.20)		
Docosapentaenoic acid C22:5n3 (g)	0.04 (0.02, 0.06)		
Docosahexaenoic acid C22:6n3 (g)	0.31 (0.18, 0.51)	+0.10–0.20 *[d]	
EPA + DHA (g)	0.40 (0.22, 0.73)	0.25 *	
Cholesterol (g)	309.2 (268.00, 376.00)		ALAP
Cholesterol (mg/1000 Kcal)	138.68 (121.54, 164.30)		

Thiamine (B_1) (mg)	1.97 (1.51, 2.44)	0.1 mg/MJ	1.4 mg
Riboflavin (B_2) (mg)	2.60 (1.83, 3.16)	2.0	1.6
Niacin (B_3) (mg)	41.62 (35.90, 49.68)	1.6 mg/MJ [e]	17 mg [e]
Pantothenic acid (B_5) (mg)	7.20 (5.68, 10.26)	7 *	7 *
Pyridoxine (B_6) (mg)	2.72 (2.24, 3.72)	1.7	2
Biotin (B_7) (μg)	46.62 (32.06, 72.72)	45 *	35 *
Folate food + folic acid (B_9) (μg)	438.20 (322.80, 612.40)	500 [f]	500 [g]
Cobalamin (B_{12}) (μg)	6.30 (5.12, 8.26)	5 *	2.8
Vitamin C (mg)	166.00 (118.38, 234.60)	155	<19y: 115<br>≥19y: 120
Vitamin A (μg)	1239.60 (964.40, 1812.60)	1300 [h]	<19y: 1200 [i]<br>≥19y: 1300 [i]
Vitamin D (μg)	4.96 (2.38, 7.38)	15 *[j]	15 [jk]
Vitamin E (μg)	15.72 (11.82, 22.40)	11 *[l]	19
Iodine (μg)	243.50 (150.40, 330.80)	200 *	290
Calcium (mg)	1112.20 (877.00, 1336.20)	18–24y:1000<br>≥25y: 950	<19y: 1300<br>≥19y: 1000
Phosphorus (mg)	1665.80 (1370.60, 1885.40)	550 *	<19y: 1250<br>≥19y: 700
Iron (mg)	19.52 (14.88, 28.84)	16	<19y: 10<br>≥19y: 9
Zinc (mg)	13.18 (10.58, 18.80)	10.4–15.6 [m]	<19y: 13<br>≥19y: 12
Selenium (μg)	113.44 (95.56, 135.80)	85 *	70

The quantitative variables are presented as means (standard deviations) when they followed a parametric distribution, and as medians (25th, 75th percentiles) when they followed a nonparametric distribution. [a] Recommended daily intake for lactating mothers. Adequate intake is presented with an asterisk (*) and PRI/RDA (i.e., Population Reference Intake for the EFSA values, and Recommended Dietary Allowance for the IOM values) in ordinary type. ** Reference intake range. [b] Depends on age and level of physical activity. [c] 0.83 g/kg body weight + 19 g/day from 0–6 months postpartum or + 13 g/day if >6 months postpartum. [d] In addition to the combined intakes of EPA and DHA of 0.25 g/day. [e] As the niacin equivalents (NE) (1 mg niacin = 1 mg NE = 60 mg dietary tryptophan). [f] DFE: dietary folate equivalents. For the combined intakes of food folate and folic acid, DFEs can be computed as follows: μg DFE = μg food folate + (1.7 × μg folic acid). [g] As the dietary folate equivalents (DFE). Furthermore, 1 DFE = 1 μg of folate from food = 0.6 μg of folic acid from fortified foods or from supplements taken with food = 0.5 μg of folic acid from supplements was taken on an empty stomach. [h] RE: retinol equivalents, 1 μg RE equals 1 μg of retinol, 6 μg of β-carotene, and 12 μg of other provitamin A carotenoids. [i] As retinol activity equivalents (RAEs). 1 RAE = 1 μg of retinol, 12 μg of β-carotene, 24 μg of α-carotene, or 24 μg of β-cryptoxanthin. [j] Assuming minimal cutaneous synthesis. In the presence of an endogenous cutaneous synthesis of vitamin D, the dietary vitamin D requirements are lower or even zero. Further, 1 μg of vitamin D ingested = 40 International Units (IU) and 0.025 μg of vitamin D ingested = 1 IU. [k] As cholecalciferol. [l] Such as ∝-tocopherol, which includes RRR-∝-tocopherol, the only form naturally present in foods, as well as the synthetic 2R-isomeric forms of ∝-tocopherol, which are found in certain fortified foods and supplements. [m] Depending on the level of a phytate intake; the higher the phytate intake, the higher the zinc requirement. Abbreviations: EFSA, European Food Safety Authority; PRI, population reference intake; AI, adequate intake; IOM, Institute of Medicine; RDA, recommended dietary allowances; Kcal, kilocalories; ALAP: as low as possible, while consuming a nutritionally adequate diet; PUFAs, polyunsaturated fatty acids; SFA, saturated fatty acids; MUFAs, monounsaturated fatty acids; EPA, eicosapentaenoic acid; DHA, docosahexaenoic acid; MJ: megajoule; and y, years.

Table S3. Prevalence of inadequate intakes of specific nutrients in human milk donors

**Nutrient**	**H-AR *** [48]	**Donors (*n* = 113), n (%)**
Thiamine (B_1), mg	1.2	7 (6.2%)

Riboflavin (B_2), mg	1.7	18 (15.9%)
Niacin (B_3), mg	13	0 (0.0%)
Pantothenic acid (B_5), mg	5.6	27 (23.9%)
Pyridoxine (B_6), mg	1.4	2 (1.8%)
Biotin (B_7), µg	36	39 (34.5%)
Folate food + folic acid (B_9), µg	380 (DFE)	44 (38.9%)
Cobalamin (B_{12}), µg	2.4	0 (0.0%)
Vitamin C, mg	145	39 (34.5%)
Vitamin A, µg RAE	1020	37 (32.7%)
Vitamin D, µg	10	99 (87.6%)
Vitamin E, mg	16	58 (51.3%)
Iodine, µg	209	40 (43.5%)
Calcium, mg	860 (19–30 y)<br>750 (31–50 y)	12 (10.6%)
Phosphorous, mg	580	0 (0.0%)
Selenium, µg	59	2 (1.8%)

[1] The number and percentage of women with inadequate intakes of each nutrient (below harmonized average requirements) are presented. * The H-AR, the harmonized average requirement, was proposed by Allen et al. (2020) [48], after they selected values from the standards set by EFSA (for Europe) and the IOM (for the United States and Canada), giving priority to those published most recently. Abbreviations: DFE, dietary folate equivalents; RAE, retinol activity equivalents; and y, years.

Table S4. Diet survey: five-day dietary diaries. Number of food servings per day consumed by the participants, healthy eating index (HEI), and records of supplement and iodized salt intake among the human milk donors

	**Donors (*n* = 113)**	**Recommendations** [49] [a]
Servings per day:		
Milk and dairy products	2.38 (1.62, 3.46)	≥4 [b]
Grains, legumes, and nuts	5.90 (4.68, 7.12)	≥7
Vegetables and greens	3.22 (2.38, 4.22)	≥4
Fruits	1.82 (1.04, 2.46)	≥3
Eggs, meat, and fish	2.84 (2.12, 3.48)	2–3 [c]
HEI *	63.67 (8.96)	
Supplement intake: yes, n (%)	60 (53.1%)	
Iodized salt intake: yes, n (%)	86 (76%)	
Salt (g/day)	1.0 (0.71, 1.34)	

The quantitative variables are presented as means (standard deviations) when they followed a parametric distribution, and as medians (25th, 75th percentiles) when they followed a nonparametric distribution. The qualitative variables are expressed as the absolute and relative frequencies (%). [a] Number of recommended daily servings of food for lactating women. [b] Preferably skimmed or semi-skimmed. [c] Preferably fat-free or very low-fat. Abbreviations: HEI, Health Eating Index. * The HEI was categorized as follows: >80 excellent, 71–80 very good, 61–70 good, 51–60 acceptable, and 0–50 inadequate [50].

Table S5. Diet survey: food frequency questionnaire (FFQ, the number of food servings per day or week consumed by the participants) results for the human milk donors

	**Donors (*n* = 113)**	**Serving Size** [51] [a]
Milk (servings/day)	1.33 (0.89, 2.22)	200–250 mL
Other dairy products (servings/day)	1.03 (0.38, 1.51)	Yogurt 200–250 g<br>Fresh cheese 80–125 g<br>Cured cheese 40–60 g

	Donors (*n* = 113)	Serving Size [51] [a]
Meats and derivatives (servings/day)	0.58 (0.37, 0.87)	100–125 g
Fish (servings/week)	2.17 (0.93, 2.96)	125–150 g
Eggs (servings/week)	2.50 (1.75, 3.50)	60 g
Fruits (servings/day)	1.91 (1.08, 3.75)	120–200 g
Raw vegetables (servings/day)	0.52 (0.21, 0.86)	150–200 g
Cooked vegetables (servings/day)	0.71 (0.43, 1.14)	150–200 g
Legumes (servings/week)	1.14 (0.71, 1.71)	60–80 g
Bread (servings/day)	1.20 (0.75, 2.40)	40–60 g
Pasta, rice, other grains (servings/week)	2.21 (1.14, 3.71)	60–80 g
Nuts (servings/week)	3.25 (1.00, 6.84)	25 g
Oils and fats (servings/day)	2.00 (1.50, 3.00)	10 g
Sweets (grams/week)	191.75 (60.00, 300.00)	

The quantitative variables are presented as medians (25th, 75th percentiles) because they all followed a nonparametric distribution. [a] Serving weight for each food group.

Table S6. Erythrocyte and plasma fatty acid compositions (g/100 g fat) for the human milk donors

Fatty Acid (%)	Common Name	Donors	Reference values [52][1]
	ERYTHROCYTES	*n* = 113	
	Saturated Fatty Acids (SFAs)		
C14:0	Myristic	0.13 (0.05)	0.51 ± 0.19
DMA C16:0	Dimethylacetal C16:0	2.18 (0.23)	
C16:0	Palmitic	21.33 (2.00)	26.54 ± 5.59
DMA C18:0	Dimethylacetal C18:0	3.43 (0.34)	
C18:0	Stearic	20.14 (19.10, 21.00)	15.61 ± 3.33
C24:0	Lignoceric	2.28 (1.63, 2.82)	5.76 ± 1.44
	Monounsaturated Fatty Acids (MUFAs)		
C17:1	Margaroleic	0.32 (0.27, 0.41)	
C18:1 *cis*-11 (n7)	Cis vaccenic	0.24 (0.07)	1.05 ± 0.18
C18:1 *cis*-9 (n9)	Oleic	12.59 (1.47)	13.30 ± 1.76
	n-6 Polyunsaturated Fatty Acids (*n*-6 PUFAs)		
C18:2 (n6)	Linoleic	8.20 (1.49)	7.52 ± 2.49
C20:3 (n6)	Dihomo-γ-linolenic	0.96 (0.68, 1.22)	1.12 ± 0.56
C20:4 (n6)	Arachidonic	24.20 (3.08)	6.88 ± 4.15
	n-3 Polyunsaturated Fatty Acids (*n*-3 PUFAs)		
C20:5 (n3)	Eicosapentaenoic	0.00 (0.00, 0.23)	0.21 ± 0.20
C22:5 (n3)	Docosapentaenoic	0.65 (0.47, 0.94)	0.89 ± 0.69
C22:6 (n3)	Docosahexaenoic	2.93 (1.19)	3.24 ± 2.46
	Fatty Acid Families		
	DMAs	5.61 (0.49)	
	SFAs	44.01 (2.63)	61.96 ± 58.12
	MUFAs	13.17 (1.52)	25.05 ± 19.03
	PUFAs	37.33 (34.99, 39.22)	13.22 ± 10.17
	MCFAs (C8-C15)	0.13 (0.05)	
	LCFAs (C16-C18)	62.88 (4.35)	
	VLCFAs (C20-C24)	29.02 (3.66)	
	n-6 PUFAs	33.44 (2.86)	
	n-3 PUFAs	3.78 (1.58)	
	n-6 PUFAs/*n*-3 PUFAs	9.38 (7.10, 12.30)	
	PLASMA	*n* = 112	
	Saturated Fatty Acids (SFAs)		

C14:0	Myristic	0.27 (0.21, 0.34)	1.13 ± 0.45
C15:0	Pentadecylic	0.05 (0.04, 0.06)	0.22 ± 0.07
DMA C16:0	Dimethylacetal C16:0	0.19 (0.14, 0.24)	
C16:0	Palmitic	21.22 (20.08, 22.25)	25.74 ± 1.98
DMA C18:0	Dimethylacetal C18:0	0.07 (0.05, 0.11)	
C18:0	Stearic	6.21 (5.84, 6.70)	5.24 ± 0.85
	Monounsaturated Fatty Acids (MUFAs)		
C16:1 *cis*-9 (n7)	Palmitoleic	0.43 (0.30, 0.54)	2.51 ± 0.79
C18:1 *cis*-11 (n7)	Cis vaccenic	0.41 (0.32, 0.54)	1.60 ± 0.25
C18:1 *cis*-9 (n9)	Oleic	17.94 (16.51, 19.64)	21.48 ± 2.70
	n-6 Polyunsaturated Fatty Acids (*n*-6 PUFAs)		
C18:2 (n6)	Linoleic	38.99 (35.78, 42.46)	24.23 ± 3.57
C20:3 (n6)	Dihomo-γ-linolenic	1.06 (0.72, 1.50)	
C20:4 (n6)	Arachidonic (ARA)	11.17 (3.17)	5.40 ± 1.35
	n-3 Polyunsaturated Fatty Acids (*n*-3 PUFAs)		
C20:5 (n3)	Eicosapentaenoic (EPA)	0.14 (0.00, 0.36)	0.32 ± 0.25
C22:6 (n3)	Docosahexaenoic (DHA)	0.72 (0.46, 1.07)	2.36 ± 0.68
	Fatty Acid Families		
	DMAs	0.27 (0.19, 0.35)	
	SFAs	27.93 (26.52, 29.15)	
	MUFAs	19.94 (16.51, 19.64)	
	PUFAs	52.83 (50.26, 55.19)	
	MCFA (C8-C15)	0.31 (0.25, 0.39)	
	LCFA (C16-C18)	85.98 (83.13, 88.49)	
	VLCFA (C20-C24)	13.41 (3.85)	
	n-6 PUFAs	51.60 (48.28, 54.09)	
	n-3 PUFAs	0.88 (0.51, 1.41)	
	n-6 PUFAs/*n*-3 PUFAs	56.49 (35.22, 105.56)	

The quantitative variables are presented as means (standard deviations) when they followed a parametric distribution, and as medians (25th, 75th percentiles) when they followed a nonparametric distribution. Abbreviations: DMA, dimethylacetal, MCFA, medium-chain fatty acids; LCFA, long-chain fatty acids; and VLCFA: very-long-chain fatty acids. [1] Data are presented as the mean ± standard deviation, from 264 lactating mothers within 72 h after delivery.

Table S7. Erythrocyte, plasma, and urine concentrations of nutrients and biochemical determinations for the human milk donors

**Variable** [1]		**Donors (*n* = 113)**	**Comments about the Reference Values or Studies in which the Corresponding Vitamers are Determined** [3]
	n	**Value** [2]	
		**ERYTHROCYTES**	
Hemoglobin (Drabkin colorimetric method) g/dL	114	25.56 (23.82, 27.57)	
EGRAC (assay kit)	93	1.23 (0.24)	Normal: < 1.2 [53,54]
Riboflavin insufficiency/deficiency (EGRAC ≥1.4) [40,53,54]		26 (28.0%)	
Marginal riboflavin status (EGRAC 1.2 to <1.4) [53,54]		24 (25.8%)	
Acceptable riboflavin status (EGRAC <1.2) [53,54]		43 (46.2%)	
Riboflavin, B2 (UPLC-MS/MS)	113		

ng/L		746.70 (557.80, 956.80)	
nM		1.98 (1.48, 2.53)	
ng/g Hb		2.93 (2.01, 3.98)	
Riboflavin deficiency (<170 nM) [53]		113 (100%)	
Nicotinamide, B3 (UPLC-MS/MS)	113		
mcg/L		5114.00 (1222.70, 6690.60)	
mcM		38.68 (9.25, 50.61)	
mcg/g Hb		18.97 (5.47, 26.45)	
Pantothenic acid, B5 (UPLC-MS/MS)	113		
mcg/L		27.50 (17.10, 60.60)	
nM		125.00 (77.73, 275.45)	
ng/g Hb		112.03 (62.32, 210.67)	
Pyridoxamine, B6 (UPLC-MS/MS)	113		
mcg/L		532.90 (411.90, 659.20)	
mcM		3.13 (2.42, 3.88)	
mcg/g Hb		1.97 (1.53, 2.69)	
		**PLASMA**	
Thiamin, B1 (UPLC- MS/MS)	113		
mcg/L		0.31 (0.19, 0.56)	
nM		1.17 (0.71, 2.11)	Reference range: 4–15 nM [55]
Riboflavin, B2 (UPLC-MS/MS)	113		
mcg/L		16.83 (12.96, 24.61)	
nM		44.58 (34.33, 65.19)	Reference interval: 6.7–50.1 nM [56]
Riboflavin <6.7 nM [56]		0 (0.0%)	
Nicotinamide, B3 (UPLC-MS/MS)	113		
mcg/L		3.88 (3.07, 5.63)	
nM		29.35 (23.22, 42.59)	Median (25th, 95th percentiles): 396.41 (264.47, 644.74) nM. A total of 1150 Australian adult women aged 43.6 (4.8) years; UHPLC/MS-MS [57]
Pantothenic acid, B5 (UPLC-MS/MS)	113		
mcg/L		123.58 (97.54, 154.69)	
nM		561.73 (443.36, 703.14)	Median (25th, 95th percentiles): 175.96 (138.79, 233.43) nM. A total of 1150 Australian adult women aged 43.6 (4.8) years; UHPLC/MS-MS [57]
Pyridoxine, B6 (UPLC-MS/MS)	113		
mcg/L		133.67 (115.44, 161.67)	
nM		726.94 (573.76, 909.94)	
Pyridoxamine, B6 (UPLC-MS/MS)	113		
mcg/L		261.48 (242.62, 282.80)	
nM		1538.12 (1427.18, 1663.53)	
Folic acid, B9 (UPLC-MS/MS)	113		
mcg/L		2.39 (1.66, 3.27)	
nM		5.41 (3.75, 7.40)	
Cobalamin, B12 (Competitive immunoassay).	113		Normal: > 221 pM [58]
pM		510.00 (405.00, 643.00)	
B12 depletion (148–221 pM [58]		1 (1.1%)	
B12 deficiency (<148 pM [58]		0 (0.0%)	
Severe B12 deficiency (<75 pM) [58]		0 (0.0%)	
Holotranscobalamin II (Immunoassay ELISA kit)	113		Normal range: 40–150 or 40–200 pM [58]

pM		176.9 (136.8, 223.3)	
B12 depletion (Holo-TC II < 35 pM) [59]		0 (0.0%)	
Homocysteine (enzymatic assay)	114		
mcM		10.33 (3.48)	
Homocysteine elevated (>13 mcM) [60,61]		25 (21.9%)	
Ascorbic acid (HPLC-DAD)	114		
mcM		45.00 (30.2, 65.8)	
<11 mcM: scurvy [41]		1 (0.9%)	
Retinol (HPLC with fluorescence and UV detector)	112		
mcg/dL		47.15 (38.05, 60.95)	
mcM		1.65 (1.33, 2.13)	
Vit A deficiency (Retinol <20 mcg/dL, <0.7 mcM) [62,63]		2 (1.8%)	
25(OH)D_3 (UPLC-electrospray ionization/tandem MS)	114		
ng/mL		6.60 (3.98, 9.63)	
nM		16.47 (9.94, 24.04)	
Risk for vit D inadequacy (25(OH)D 12-<20 ng/mL; 30-<50 nM) [44,64,65]		13 (11.4%)	
Risk for vitamin deficiency (25(OH)D <12 ng/mL; < 30 nM) [44,64,65]		100 (87.7%)	
1,25$(OH)_2D_3$ (UPLC-electrospray ionization/tandem MS)	114		
pg/mL		97.36 (64.64, 143.58)	
pM		233.68 (155.15, 344.61)	
Cholecalciferol (UPLC-electrospray ionization/tandem MS)	114		
pg/mL		15.10 (4.95, 76.55)	
α-tocopherol (HPLC with fluorescence and UV detector)	111		
mcg/dL		221.50 (166.30, 290.40)	
mcM		5.14 (3.86, 6.74)	
Vit E deficiency (<500 mcg/dL; <0.5 mg/dL; <11.6 mcM) [41]		100 (90.10%)	
Severe vit E deficiency (<5.8 mcM) [66]		73 (65.8%)	
α-tocopherol:total lipids (cholesterol+triacylglycerols)	111		Lower limit of the normal range: 1.6–2.4 mcmol:mmol [66]
(mcmol:mmol)		0.97 (0.67, 1.46)	
Ratio < 1.6 mcmol:mmol [66]		90 (81.1%)	
α-tocopherol:cholesterol (mcmol:mmol)	111	1.07 (0.77, 1.64)	Lower limit of the normal range: 2.2–2.5 mcmol:mmol [66]
Ratio <2.2 mcmol:mmol [66]		97 (87.4%)	
γ-tocopherol (HPLC with fluorescence and UV detector)	108		
mcg/dL		35.60 (21.70, 48.70)	
Total cholesterol (enzymatic assay)	114		
mg/dL		185.80 (163.00, 200.90)	
mM		4.81 (4.22, 5.20)	
Hypercholesterolemia (≥ 240 mg/dL) [65]		9 (7.9%)	
Triacylglycerols (enzymatic assay)	114		
mg/dL		43.70 (37.30, 56.00)	
mM		0.49 (0.42, 0.63)	
Hypertriglyceridemia (≥ 200 mg/dL) [65]		0 (0.0%)	
HDL (enzymatic assay)	114		
mg/dL		62.23 (10.03)	
mM		1.58 (0.26)	

Low HDL levels (< 40 mg/dL) [65]		1 (0.9%)	
LDL (enzymatic assay)	114		
mg/dL		104.57 (26.30)	
mM		2.70 (0.68)	
High LDL levels (≥ 160 mg/dL) [65]		4 (3.5%)	
		**URINE**	
Cr (Jaffé colorimetric kinetic method)	114		
mg/dL		121.38 (51.02)	
Methylmalonic acid (UPLC-MS/MS)	113		Normal ranges are laboratory dependent
mg/L		4.83 (3.51, 6.78)	
mcg/mg Cr		4.81 (3.40, 6.76)	
mcmol/mmol Cr		4.61 (3.26, 6.48)	Normal range: 0.0–3.6 mmol/mol [67]
B12 deficiency marker (Methylmalonic acid/Cr >4 mcg/mg; >3.8 mmol/mol) [59]		73 (64.6%)	Definite cases of vitamin B12 deficiency: 4.8 mcmol/mmol for [68]
Iodine (ICP-MS)	113		
mcg/L		111.87(73.60, 159.70)	Adequate: median > 100 mcg/L [69]
mcg/mg Cr		0.10 (0.07, 0.15)	Adequate intake: 85–220 mcg/g Cr [70]
Sodium (ICP-MS)	113		
mg/L		3354.87 (1332.99)	
M		0.15 (0.01)	
mg/mg Cr		3.00 (2.09, 3.84)	
mmol/g Cr		130.49 (9091, 167.03)	<47 mmol/g: low; 47–114 mmol/g: intermediate; >114 mmol/L: high [70]
Calcium (ICP-MS)	113		
mg/L		77.51 (44.00, 143.33)	
mg/mg Cr		0.08 (0.04, 0.12)	Normal: < 0.14 [71]
Phosphorus (ICP-MS)	113		
mg/L		953.60 (628.70, 1419.27)	
mg/mg Cr		0.84 (0.63, 1.04)	Normal range: 0.22–2.17 [72]

[1] The units of our results have been converted to the international system. The molecular weights used for unit conversion are as follows: thiamine 265 g/mol, riboflavin 377.5 g/mol, nicotinamide 132.2 g/mol, pantothenic acid 220 g/mol, pyridoxine 170 g/mol, pyridoxamine 170 g/mol, folic acid 442.1 g/mol, retinol 286.5 g/mol, 25(OH)D 400.6 g/mol, 1,25(OH)$_2$D 416.64 g/mol, α-tocopherol 430. 71 g/mol, total cholesterol 386.65 g/mol, triacylglycerols 886 g/mol, methylmalonic acid 118-091 g/mol, creatinine 113.12 g/mol, sodium 22.99 g/mol. [2] Quantitative variables are expressed as means (standard deviations) when they were distributed parametrically and as medians (25th and 75th percentiles) when they were distributed non-parametrically. Qualitative variables are presented as the absolute and relative frequencies (%). [3] In the absence of clearly established reference values, the results of studies in which the same vitamers have been determined are presented (further information is available in a previously published manuscript [36]. Abbreviations: *n*, number of samples; EGRAC, erythrocyte glutathione reductase activity coefficient: EGRA with an excess of flavine adenine dinucleotide (FAD)/EGRA under baseline conditions; UPLC, ultra-performance liquid chromatography; MS/MS, tandem mass spectrometry; Hb, hemoglobin; UHPLC, ultra-high-performance liquid chromatography; Holo-TCII, holotranscobalamin II; HPLC, high-performance liquid chromatography; DAD, diode array detector; UV, ultraviolet; MS, mass spectrometry; Cr, creatinine; ICP, inductively coupled plasma; and M, molar.
